# Impedance Responses Reveal β_2_-Adrenergic Receptor Signaling Pluridimensionality and Allow Classification of Ligands with Distinct Signaling Profiles

**DOI:** 10.1371/journal.pone.0029420

**Published:** 2012-01-05

**Authors:** Wayne Stallaert, Jonas F. Dorn, Emma van der Westhuizen, Martin Audet, Michel Bouvier

**Affiliations:** 1 Department of Biochemistry, Université de Montréal, Montréal, Quebec, Canada; 2 Institute for Research in Immunology and Cancer, Université de Montréal, Montréal, Quebec, Canada; University of Illinois at Urbana-Champaign, United States of America

## Abstract

The discovery that drugs targeting a single G protein-coupled receptor (GPCR) can differentially modulate distinct subsets of the receptor signaling repertoire has created a challenge for drug discovery at these important therapeutic targets. Here, we demonstrate that a single label-free assay based on cellular impedance provides a real-time integration of multiple signaling events engaged upon GPCR activation. Stimulation of the β_2_-adrenergic receptor (β_2_AR) in living cells with the prototypical agonist isoproterenol generated a complex, multi-featured impedance response over time. Selective pharmacological inhibition of specific arms of the β_2_AR signaling network revealed the differential contribution of G_s_-, G_i_- and Gβγ-dependent signaling events, including activation of the canonical cAMP and ERK1/2 pathways, to specific components of the impedance response. Further dissection revealed the essential role of intracellular Ca^2+^ in the impedance response and led to the discovery of a novel β_2_AR-promoted Ca^2+^ mobilization event. Recognizing that impedance responses provide an integrative assessment of ligand activity, we screened a collection of β-adrenergic ligands to determine if differences in the signaling repertoire engaged by compounds would lead to distinct impedance signatures. An unsupervised clustering analysis of the impedance responses revealed the existence of 5 distinct compound classes, revealing a richer signaling texture than previously recognized for this receptor. Taken together, these data indicate that the pluridimensionality of GPCR signaling can be captured using integrative approaches to provide a comprehensive readout of drug activity.

## Introduction

G protein-coupled receptors (GPCRs) are the most abundant class of cell surface receptors, responding to various types of endogenous stimuli, including hormones, neurotransmitters and odorants, and are the largest family of therapeutic targets [1], [2]. Through interactions with various G protein [3] and non-G protein effectors [4], GPCRs elicit a diverse array of signaling events, including production of second messengers, activation of phosphorylation cascades, modulation of ion channel activity and transcriptional regulation. Although it was originally assumed that a given GPCR controlled the activity of a single signaling pathway, it is now recognized that individual receptors can elicit multiple signaling events resulting in global changes in cellular physiology, thus highlighting the pluridimensionality of GPCR signaling [5]. Furthermore, certain GPCR ligands have been shown to differentially modulate distinct subsets of this signaling repertoire, a phenomenon referred to as “ligand-biased signaling” or “functional selectivity” [5]–[7]. A recent review of the published literature indicates that ligand-biased signaling occurs for a large number of GPCRs involved in a diverse array of physiological functions [8]. For example, several peptide ligands for the angiotensin AT_1A_ receptor show a clear preference in the activation of β-arrestin-dependent signaling events, yet possess no intrinsic efficacy towards G protein-dependent signaling [9], [10]. Even more striking is the inversion of efficacy observed for some β-adrenergic receptor ligands, which act as inverse agonists for cAMP production yet behave as agonists for the activation of the ERK1/2 MAPK pathways [11], [12]. Harnessing such ligand functional selectivity could represent a promising avenue in drug development, as the design of compounds that selectively modulate a pathway involved in a given pathology without collateral effects on other pathways could provide therapeutic benefit with a decreased risk of side effects. Indeed, several recent studies have suggested that functionally selective ligands may provide clinically relevant advantages over unbiased ligands at the same receptor [13]–[15].

Yet, detecting the full extent of such ligand “functional selectivity” remains a non-trivial technical challenge and has traditionally involved measuring the relative efficacy of ligands towards distinct pathways engaged by a given receptor using multiple assays. One considerable challenge in obtaining a full description of ligand activity at a given GPCR is the lack of knowledge about the complete signaling repertoire of most receptors. In addition, monitoring multiple signaling pathways can be time and resource consuming and involves the use of different assay formats with different sensitivity and dynamic ranges that can lead to spurious conclusions.

An integrative approach that could capture the global signaling profile of a ligand in a single assay would therefore greatly facilitate the identification of biased ligands and enable their classification into pharmacologically relevant categories. Besides the potential impact on the drug discovery process, such an approach could also provide greater insight into how the pluridimensionality of GPCR signaling is integrated into an overall cellular response. A string of recent studies has explored the use of such integrative assays to study GPCR pharmacology using label-free, cell-based technologies developed to monitor real-time changes in higher-order cellular events such as morphology, viability, adhesion and mass distribution [16]–[20]. Thus far, however, little effort has been made to understand how the pluridimensionality of GPCR signaling is recapitulated in the responses generated by these assays. One recently developed label-free assay is the measurement of cellular impedance. The use of impedance measurements to monitor global cellular activity is based on the principle that the adhesion of cells directly onto microelectrodes induces changes in the local ionic environment at the electrode/solution interface, conferring an increase in electrode impedance (see Materials and Methods). Any changes in cell morphology and/or adhesion that modulate the physical contact between cell and electrode will be reflected by changes in impedance. Recently, impedance measurements have been used to monitor GPCR signaling [16], [18], [19] and to assess general mechanisms of drug action [21].

In the present study, we sought to determine if impedance signatures represent an integrative measure of the receptor signaling repertoire that can be used to differentiate among ligands with distinct signaling profiles. Using the β_2_-adrenergic receptor (β_2_AR) as a model, we dissected the impedance responses from individual ligand-receptor pairs and associated the activation of specific signaling events with distinct features of the impedance curves. This dissection of the impedance responses led to the identification of a novel β_2_AR-mediated Ca^2+^ signaling event, highlighting the strength of such an integrative readout of cellular response to reveal biological processes even at a well-characterized target. Finally, we performed an unsupervised clustering analysis that permitted a rapid and systematic classification of compounds based on the impedance signatures obtained in both heterologous and primary cell cultures, demonstrating that impedance responses represent signatures that are predictive of the global signaling profiles of β-adrenergic ligands previously shown to display functional selectivity [10], [11].

## Results

### β_2_AR activation elicits a multi-featured, time-resolved impedance response

**Figure 1:**
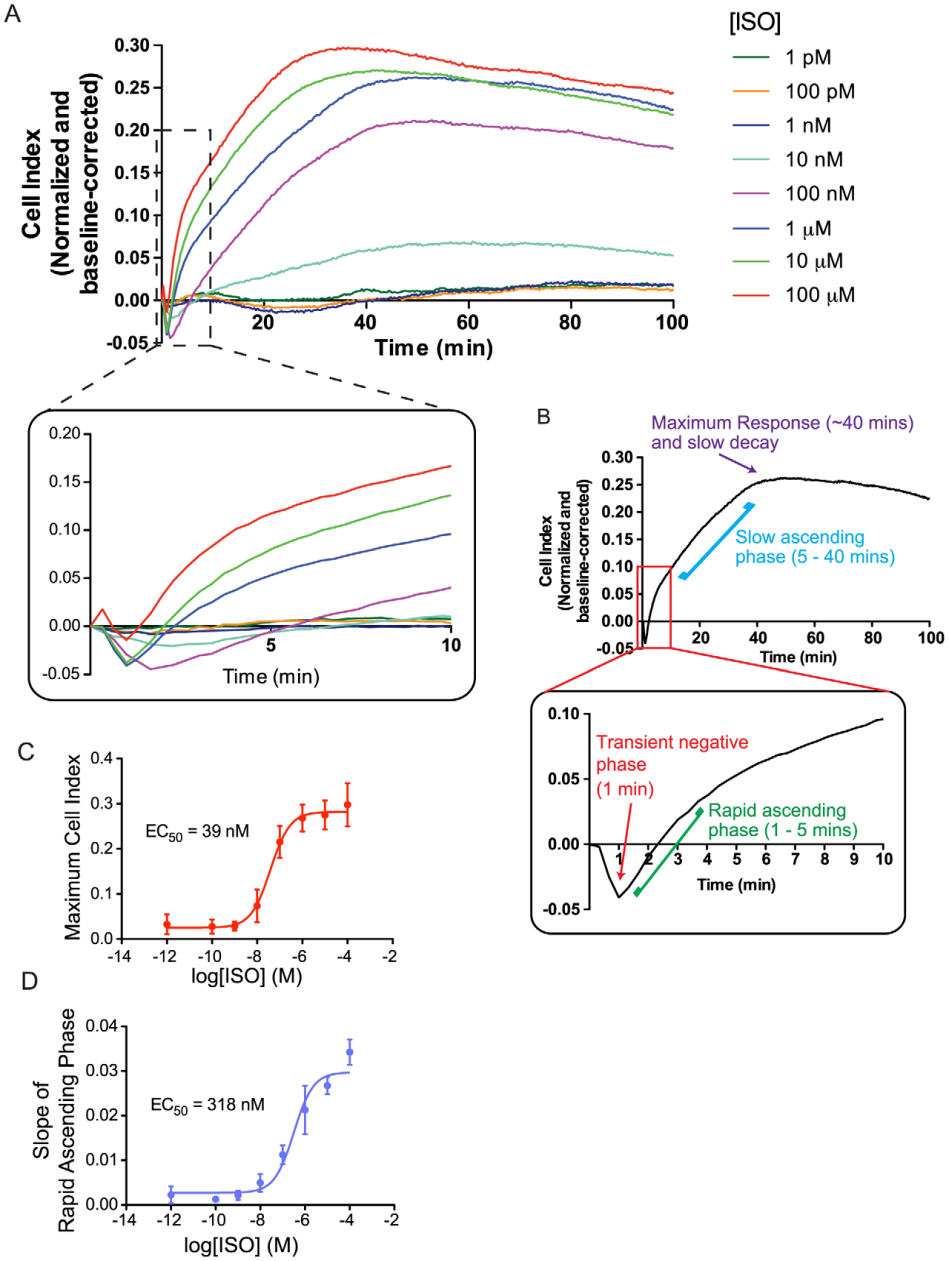
Ligand-induced changes in impedance are multi-featured and concentration-dependent. (**A**) Impedance measurements were obtained in 6HisHA-β_2_AR-HEK293S cells following treatment with isoproterenol (ISO) at the concentrations indicated. Impedance responses (represented as changes in Cell Index) were normalized and baseline-corrected as described in *Materials and Methods* and Figure S9. Inset: Enlargement of the transient negative phase of the response. (**B**) Features of the ISO impedance signature with the inset showing an enlargement of the first 10 minutes post-stimulation. (**C,D**) Concentration-response curves describing the maximum impedance response generated (**C**) and the slope of the rapid ascending phase (**D**) reveal distinct EC_50_ values. Data represent means of at least three independent experiments (± SEM for C and D). For clarity, error bars are not shown for impedance responses. Typical experimental variability in the impedance responses is demonstrated in Figure S5.

We first sought to determine if impedance measurements could provide a composite representation of the multiple signaling events elicited upon receptor activation. To assess this possibility, impedance measurements were made in HEK293S cells stably overexpressing the human β_2_AR (6HisHA-β_2_AR-HEK293S) following treatment with the prototypical β_2_AR-selective agonist isoproterenol (ISO). As shown in **Figure 1A**, stimulation of cells with ISO led to a concentration-dependent, time-resolved and feature-rich impedance response. Immediately following ISO-stimulation, the impedance response briefly decreases below zero before quickly reverting to a positive slope, producing what is referred to hereafter as the “transient negative phase” (**Figure 1B**). Subsequently, the impedance rises sharply at first (“rapid ascending phase”), followed by a later “slow ascending phase” before reaching a maximum, after which it decays slowly. This response was found to be β_2_AR-specific since the response was blocked by the selective β_2_AR antagonist ICI118,551 (ICI) but was not affected by the β_1_AR-selective antagonist CGP20712A (**Figure S1A**). In addition, the concentration-response curve describing the maximum impedance response elicited by ISO was progressively right-shifted with increasing concentrations of ICI, indicating a competitive antagonism of the response by β_2_AR blockade (**Figure S1B**). Further supporting the β_2_AR specificity of the response, we observed a qualitatively similar ICI-sensitive impedance response in the parental HEK293S cell line, which endogenously express low amounts of β_2_AR (**Figure S2**). Although the impedance response maintains the same general shape, a decrease in the magnitude of the positive component of the impedance response and a larger transient negative phase was observed.

Further analyses of the impedance responses were performed in the 6HisHA-β_2_AR-HEK293S cell line to obtain a more robust response to receptor stimulation. The distinct features of the response (i.e. depth and duration of the transient negative phase, slope of the rapid ascending phase and the maximal impedance response) were differentially modulated by increasing concentrations of ISO, suggesting the contribution of distinct signaling events to discrete components of the impedance response. For instance, concentration-response curves describing either the maximum impedance response (**Figure 1C**) or the slope of the rapid ascending phase (**Figure 1D**), revealed EC_50_ values that differ by more than an order of magnitude. Furthermore, two concentration-dependent components can also be distinguished in the transient negative phase. At low concentrations (i.e. under 1 µM), ISO promoted a concentration-dependent increase in the magnitude of the minimum (**Figure 1A**). At higher concentrations, however, no further increase in depth was observed, but a quicker recovery from negative impedance values and a shift of the minimum to earlier time points occurred, thus reducing the duration of the transient negative phase. Receptor expression was also found to differentially influence distinct components of the impedance response. As illustrated above, overexpression of the β_2_AR selectively increases the positive components of the impedance response while decreasing the duration and amplitude of the transient negative phase (**Figure S2A**). These results further support the above data that the signaling events contributimg to the transient negative phase are fully activated with fewer stimulated receptors. As the stimulus strength (i.e. agonist concentration) or receptor number is increased, the impedance response becomes more positive, likely due to the engagement of another signaling event with a weaker stimulus-coupling. Altogether, these results suggest that the impedance response obtained reflects the integration of multiple signaling events which may be individually represented in the various components of the response.

### Impedance responses encode multiple signaling events upon β_2_AR activation

**Figure 2:**
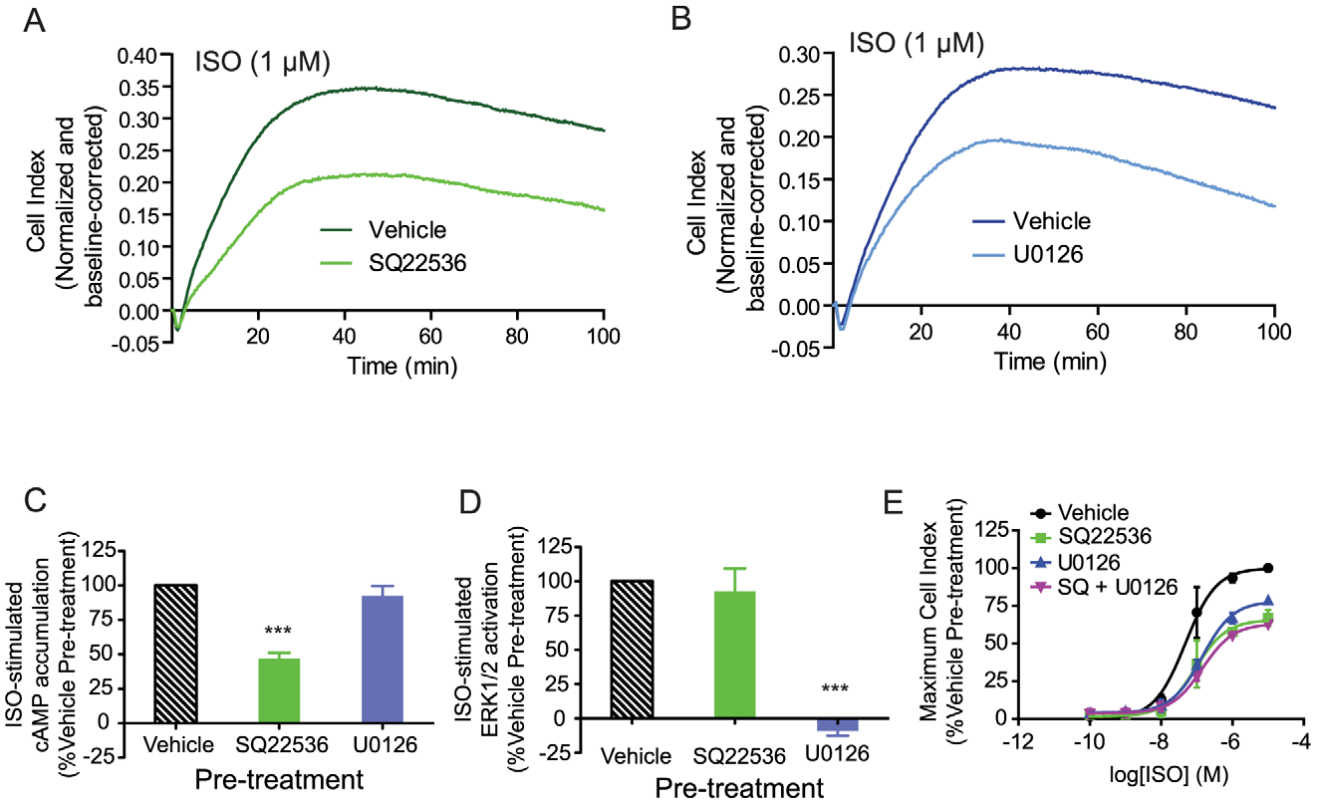
Contribution of cAMP accumulation and ERK1/2 activation to the ISO-promoted impedance response. (**A,B**) Pre-treatment for 1 hour with the adenylyl cyclase inhibitor, SQ2236 (100 µM, A), or the MEK inhibitor, U0126. (5 µM, B) leads to decreases in the kinetics of the slow ascending phase and the maximum value of ISO-promoted impedance response. (**C,D**) SQ22536 and U0126 selectively inhibit ISO-promoted cAMP accumulation (C) and ERK1/2 activation (D), respectively. (**E**) Concurrent inhibition of cAMP and ERK1/2 pathways with SQ22536 and U0126 does not lead a further attenuation of the ISO-promoted maximum impedance response than either inhibitor alone. Data represent means of at least three independent experiments (± SEM for C–E). For statistical analysis, individual conditions were compared in **C** and **D** using a one-way ANOVA and a Bonferroni post-hoc test. *** P<0.001.

β_2_AR activation is known to elicit at least two distinct signaling events in HEK293S cells: cAMP accumulation via adenylyl cyclase (AC) stimulation and activation of the ERK1/2 MAPK pathway [12]. To directly investigate if these canonical pathways are represented in the impedance response, 6HisHA-β_2_AR-HEK293S cells were pre-treated, prior to ISO stimulation, with the AC inhibitor SQ22536 to inhibit cAMP production or the MEK inhibitor U0126 to block ERK1/2 activation. Interestingly, both of these inhibitors led to similar decreases in the slope of the slow ascending phase and the maximum impedance response without affecting the transient negative phase or the rapid ascending phase (**Figure 2A,B**). A similar effect on the impedance response was observed using the K97A dominant negative mutant of MEK1 (**Figure S3**). SQ22536 and U0126 were found to be pathway-specific since they blocked the ISO-stimulated accumulation of cAMP and activation of ERK1/2, respectively, and had no significant effects on the other signaling pathway (**Figure 2C,D**). The observation that cAMP and ERK1/2 pathway inhibition led to similar changes in the impedance response suggested that these pathways may converge on a common mechanism underlying the observed changes in impedance. Consistent with this notion, concurrent inhibition of both pathways did not lead to a greater attenuation of the maximal ISO-stimulated impedance response than inhibition of either the cAMP or ERK1/2 pathway alone (**Figure 2E**).

**Figure 3:**
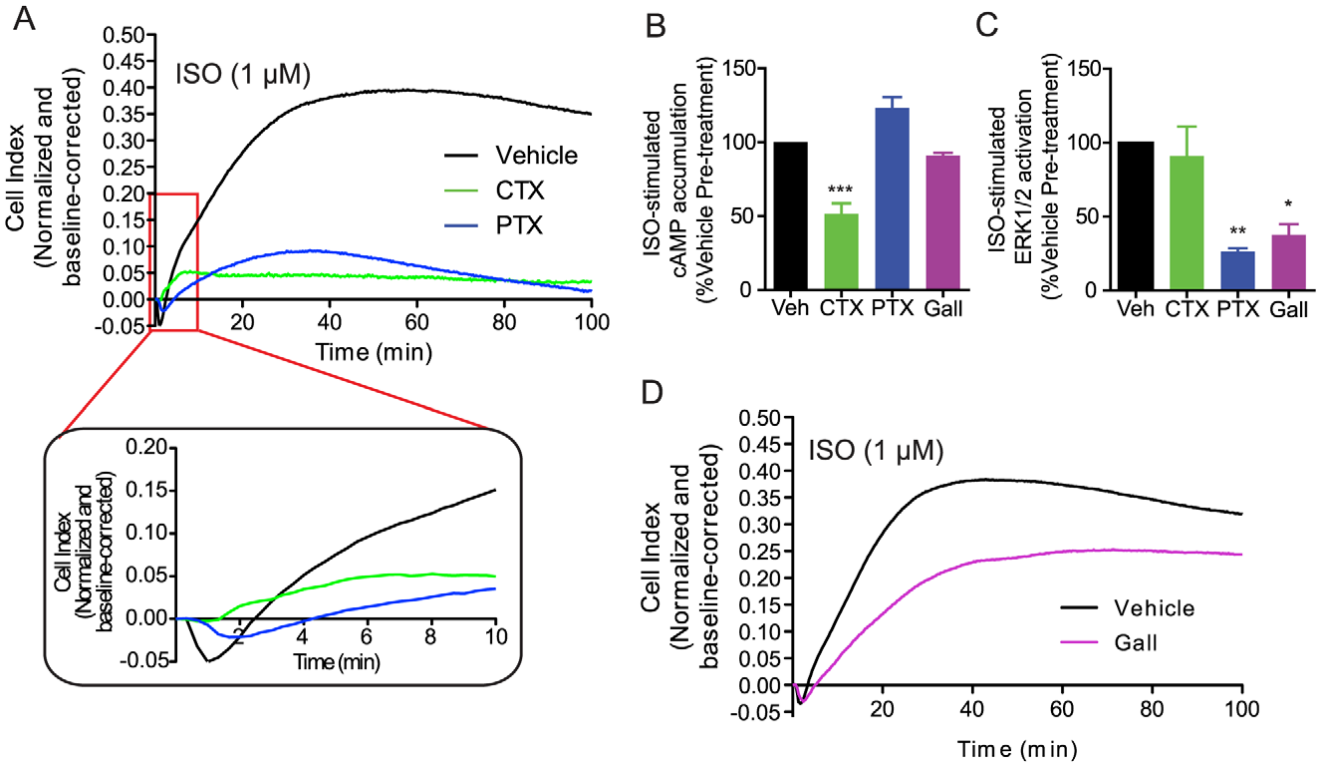
Contribution of G_S_, G_i_, and Gβ/γ to the ISO-promoted impedance response. (**A**) Inhibition of G_S_- and G_i_-dependent signaling by 16 hour treatment with cholera toxin (CTX, 200 ng/ml) and pertussis toxin (PTX, 100 ng/ml), respectively, leads to distinct changes in the ISO-promoted impedance response. Inset: Enlargement of the transient negative phase of the response. (**B,C**) Effects of CTX, PTX or gallein (Gall, 100 µM, 1 hour pre-treatment) on ISO-promoted cAMP (B) and ERK1/2 responses (C). (**D**) Inhibition of Gβγ-dependent signaling by Gall leads to decreases in the kinetics of the slow ascending phase and maximum value of ISO-promoted impedance response. Data represent means of at least three independent experiments (± SEM for B and C). For statistical analysis, individual conditions were compared in **B** and **C** using a one-way ANOVA and a Bonferroni post-hoc test. *** P<0.001, ** P<0.01, and * P<0.05.

From the above data, it is clear that cAMP production and MAPK activation are not sufficient to explain the entire ISO impedance response since a considerable residual signal is observed when both of these pathways are inhibited. To further explore the source of this residual response, we examined the role of the G proteins themselves. The β_2_AR is known to couple to both G_s_ and G_i_ [22]. Chronic treatment with cholera toxin (CTX), which selectively downregulates G_s_ [23], led to a severe attenuation of the impedance response, including a complete loss of the transient negative phase and a significant reduction of the maximum response (**Figure 3A**). Interestingly, CTX inhibited ISO-induced cAMP accumulation to a similar extent as the AC inhibitor SQ22536, with no effect on ERK1/2 activation (**Figure 3B,C**), yet the effect of CTX on the impedance response is clearly distinct from the one observed following inhibition of the cAMP pathway (**Figure S4A**), suggesting the contribution of additional G_s_-dependent signaling events other than cAMP accumulation to the impedance response. Selective inhibition of G_i_-dependent signaling with pertussis toxin (PTX) led to changes in the impedance response that were different from those caused by CTX (**Fig. 3A**). Specifically, a transient negative phase was still observed following PTX treatment, although it was reduced and delayed compared to vehicle treatment. Although both treatments led to significant reductions in the maximum impedance response, the influence of PTX on this phase of the curve was not as severe as CTX. Taken together, these results indicate that G_s_ and G_i_ signaling have distinguishable contributions to the overall impedance response. PTX treatment significantly inhibited ERK1/2 activation but did not affect cAMP production (**Figure 3B,C**). However, the contribution of G_i_ cannot be solely attributed to ERK1/2 activation since complete abolition of this pathway by U0126 (**Fig. 2D**) affected the ISO-stimulated impedance response to a lesser extent than PTX (**Figure S4B**), which only partially blocked ERK1/2 activation. Although the differences observed between acute pharmacological inhibition of cAMP production and ERK1/2 activation versus chronic inhibition of Gs and Gi suggest the contribution of alternative G protein-dependent signaling pathways to the impedance response, we cannot exclude the possibility that the chronic treatment with CTX and PTX may affect other downstream components involved in the response.

Next, we assessed the contribution of Gβγ subunits to the impedance response using the pharmacological inhibitor, gallein, which selectively inhibits interactions between Gβγ and effector molecules [24]. Pre-treatment with gallein inhibited ERK1/2 activation, with no effect on cAMP accumulation (**Figure 3B,C**), and led to an impedance response with reduced kinetics of the slower ascending phase and a decreased maximum response (**Figure 3D**), The effect of gallein was similar overall to that observed upon inhibition of the ERK1/2 pathway; however, Gβγ inhibition led to an additional loss of the slow decay phase (**Figure S4C**) indicating the existence of additional Gβγ-dependent signaling beyond ERK1/2.

Altogether, these data demonstrate that a variety of G protein-dependent signaling events are integrated in the ISO-stimulated impedance response, including the activation of canonical second messenger pathways (i.e. cAMP and ERK1/2) and additional G protein-dependent events not yet identified.

### Clustering analysis of impedance signatures reveals compound classes with distinct signaling profiles

**Figure 4:**
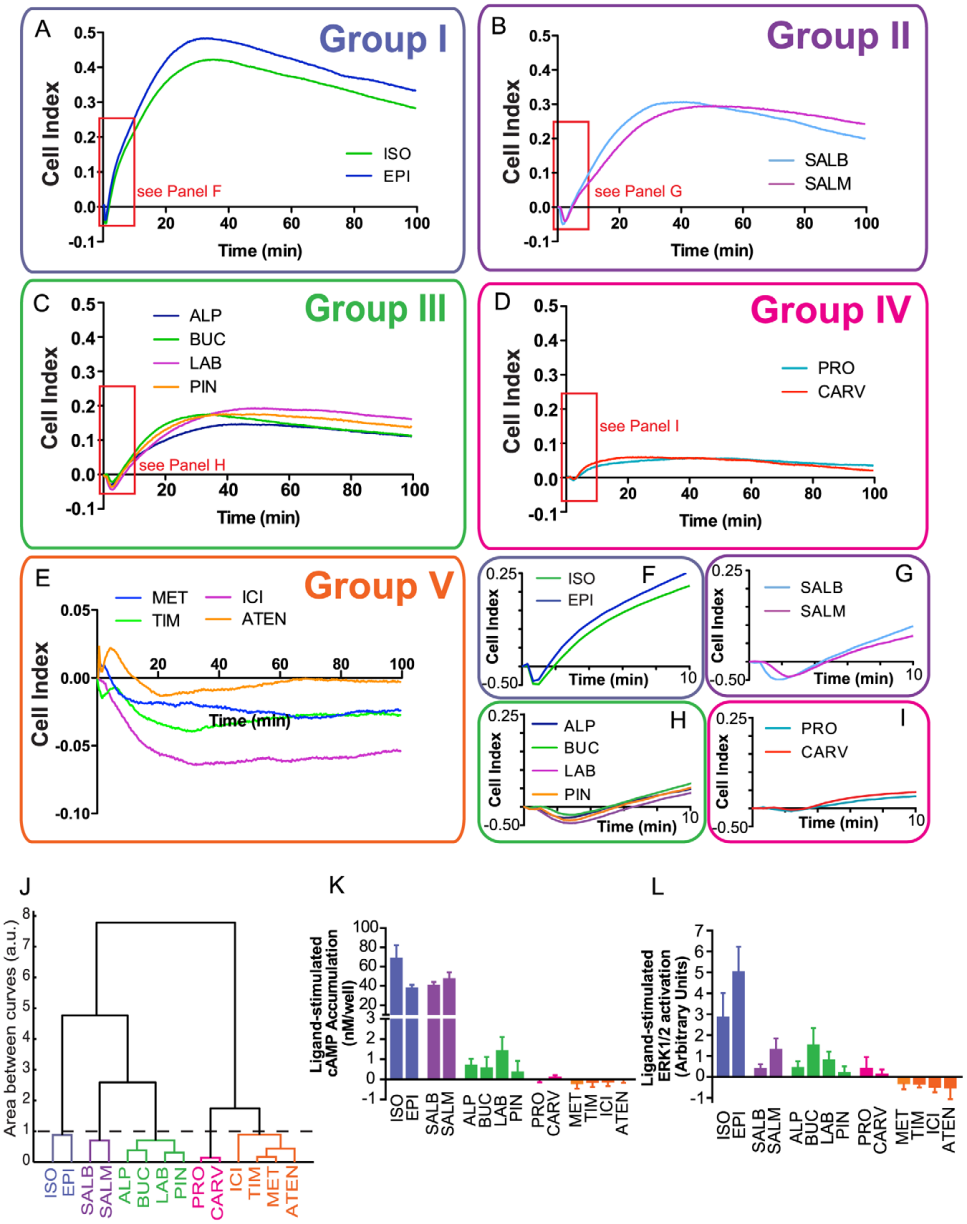
Impedance signatures reveal 5 distinct compound classes among β-adrenergic ligands. (**A–E**) Impedance responses were obtained following stimulation with saturating concentrations of the following ligands: ISO (50 µM), epinephrine (EPI, 75 µM), salbutamol (SALB, 100 µM), salmeterol (SALM, 1 µM), alprenolol (ALP, 1 µM), bucindolol (BUC, 1 µM), labetalol (LAB, 1 µM), pindolol (PIN, 1 µM), propranolol (PRO, 1 µM), carvedilol (CARV, 1 µM), metoprolol (MET, 20 µM), timolol (TIM, 1 µM), ICI118,551 (ICI, 1 µM) and atenolol (ATEN, 100 µM). Impedance responses, as represented by the Cell Index, were normalized and baseline-corrected. The first 10 minutes of the impedance responses are enlarged in panels **F–I**. (**J**) Ligands were categorized according to complete linkage hierarchical clustering (see *Materials and Methods*) determined by comparing the area between individual curves. The dashed line represents the calculated threshold value defining compound dissimilarity. (**K,L**) Efficacies of compounds for cAMP (K) and ERK1/2 (L) pathways performed in conditions identical to those used for impedance measurements in (A–E). Data represent means of at least three independent experiments (± SEM for K and L).

After demonstrating that impedance responses represent the integration of multiple signaling events elicited upon receptor activation, we hypothesized that compounds with different signaling profiles at the β_2_AR would generate distinct impedance signatures. Impedance responses were obtained for a series of β-adrenergic ligands (**Figures 4A–I** **and** **S5**) at concentrations providing >99% receptor occupancy based on their reported *K_d_* values [25]–[27]. Differences among ligand signatures were determined by comparing the area between individual impedance curves. Visualization of the pairwise differences using a visual assessment of clustering tendency (VAT; see *Materials and Methods*) suggested that the ligands fall into 5 distinct clusters (**Figure S6**), which we confirmed by complete linkage hierarchical clustering [28] (**Figure 4J**). With the exception of Group V compounds, which elicit impedance responses of completely negative values, differences between compound classes reflect differences in the specific features defined in our previous analysis of the ISO-promoted impedance response. We see great variation, for example, in the maximum impedance responses generated between compound classes. We also observed clear distinctions between groups in the transient negative phase of the response; Group I compounds elicit a relatively short-lived transient negative phase while Group IV compounds completely lack this feature (**Figure 4F–I**).

To determine the relationship between ligand signaling profiles with the compound classes obtained in the above analysis of impedance signatures, we assayed each of the ligands for their ability to stimulate cAMP accumulation and ERK1/2 activation in conditions identical to those used for the impedance measurements (**Figure 4K,L**). Group I ligands, which include ISO, have high agonist efficacies (full or strong partial agonists) for both cAMP and ERK1/2 responses. Group II ligands are only weak partial agonists toward the ERK1/2 pathway but elicit as strong cAMP responses as Group I. Group III ligands are weak partial agonists for both cAMP and ERK1/2 pathways. Group IV ligands elicit very weak signaling responses for both pathways. Group V ligands are inverse agonists towards both cAMP and ERK1/2 responses. The compound classes defined by impedance signatures are thus highly predictive of the relative efficacies of ligands to elicit cAMP accumulation and ERK1/2 activation.

### The role of intracellular Ca^2+^ in the β_2_AR-mediated impedance response

**Figure 5:**
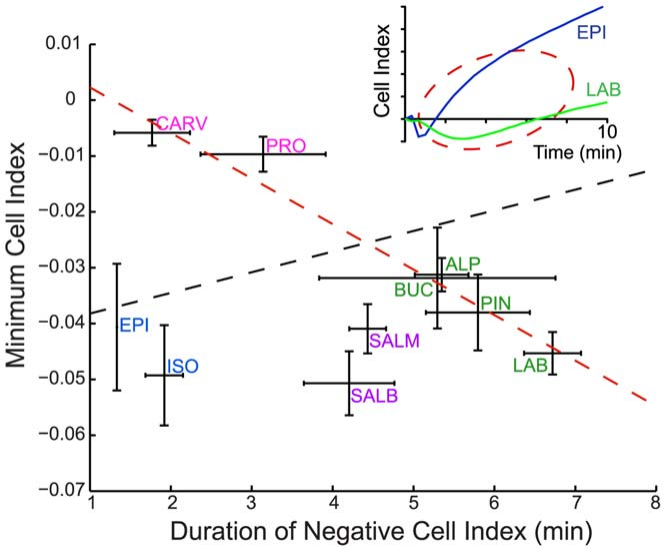
Analysis of the transient negative phase of the impedance response reveals differences between Group I and II vs Group III and IV ligands. The duration of the negative phase was plotted as a function of the minimum value for all ligands promoting a transient negative response (Group I–IV). Compound classes are indicated by color: Group I – blue, Group II – purple, Group III – green, and Group IV – pink. Lines of best fit were determined using weighted total least squares regression for all ligands (black dotted line, r^2^ = 0.06, P = 0.79) and for Group III and IV compounds only (red dotted line, r^2^ = 0.97, P = 9.0×10^−5^). Inset: The comparison of transient negative phases promoted by the prototypical Group I and Group III ligands EPI (dark blue) and LAB (green), respectively. Data represent means of at least three independent experiments (± SEM).

**Figure 6:**
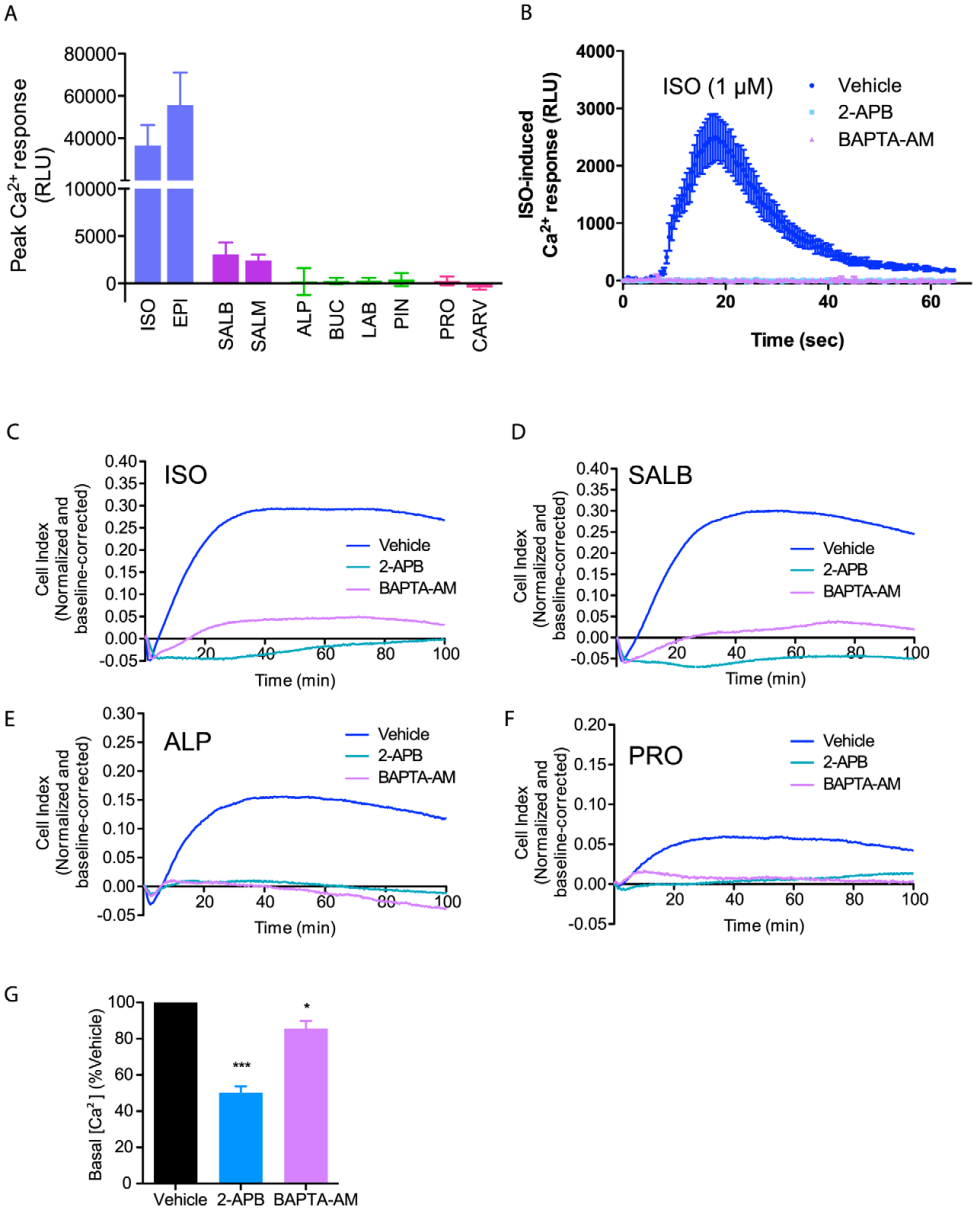
The role of intracellular Ca^2+^ in the impedance response to β_2_AR stimulation. (**A**) Peak Ca^2+^ responses generated upon stimulation with the indicated ligand (1 µM each). The intracellular Ca^2+^ concentration is represented in relative luminescence units (RLU) emitted by the obelin biosensor (see *Materials and Methods*). (**B**) Pre-treatment for 1 hour with the IP_3_ receptor antagonist 2-aminoethoxydiphenyl borate (2-APB, 200 µM) or the intracellular Ca^2+^ chelator BAPTA-AM (20 µM) completely abolishes the ISO-promoted Ca^2+^ response. (**C–F**) Pre-treatment with 2-APB or BAPTA-AM also leads to an inhibition of the rapid ascending phase and a decrease in the maximum impedance responses of ligands from Groups I to IV: ISO (C), salbutamol (SALB, D), alprenolol (ALP, E) and propranolol (PRO, F), respectively (1 µM each). (**G**) Pre-treatment with 2-APB or BAPTA-AM significantly decreases basal intracellular [Ca^2+^]. Data represent means of at least three independent experiments (± SEM for A, B and G). For statistical analysis, individual conditions were compared in **G** using a one-way ANOVA and a Bonferroni post-hoc test. *** P<0.001 and * P<0.05.

Since neither cAMP accumulation nor ERK1/2 activation contribute to the transient negative phase observed for most ligand impedance signatures, we reasoned that further analysis of this feature could reveal additional texture among ligand signaling profiles. The shape of the transient negative phase can be defined by two parameters, the duration and minimum value of the negative response. Although no significant correlation between these two parameters could be observed when all of the ligands were considered (r^2^ = 0.06, p = 0.79) (**Figure 5**), a strong linear relationship emerged when considering Groups III and IV compounds alone (r^2^ = 0.97, p = 9×10^−5^), reflecting a fundamental difference in the nature of the transient negative phase between Group I/II ligands and Group III/IV ligands. Indeed, Group I and II ligands have a shorter-lived transient negative phase than would be predicted given their amplitudes (**Figure 5** **inset**), suggesting that these compounds may selectively engage a signaling pathway that accelerates the rapid ascending phase thus shortening the duration of the transient negative phase. Given that many GPCRs are capable of activating pathways leading to an increase in intracellular [Ca^2+^], we assessed whether a β_2_AR-mediated Ca^2+^ response could be responsible for accelerating this phase. Consistent with this hypothesis, only Group I and II ligands elicited a significant increase in intracellular [Ca^2+^], with ISO and epinephrine (Group I) eliciting a much stronger Ca^2+^ response than salbutamol and salmeterol (Group II) (**Figure 6A**). This response was found to be β_2_AR-selective since receptor blockade with the β_2_AR-selective antagonist ICI completely abolished the ISO-stimulated increase in intracellular [Ca^2+^] (**Figure S7**). This β_2_AR-promoted increase in [Ca^2+^] was sensitive to the inositol trisphosphate (IP_3_) receptor antagonist, 2-aminoethoxydiphenyl borate (2-APB) and the intracellular Ca^2+^ chelator BAPTA-AM (**Figure 6B**), implicating a mobilization from IP_3_-gated intracellular stores in the calcium response. Pre-treatment with either 2-APB or BAPTA-AM strongly inhibited the ascending phases of the impedance response (**Figure 6C**), supporting the hypothesis that Ca^2+^ mobilization is a key event in the β_2_AR-mediated impedance response.

**Figure 7:**
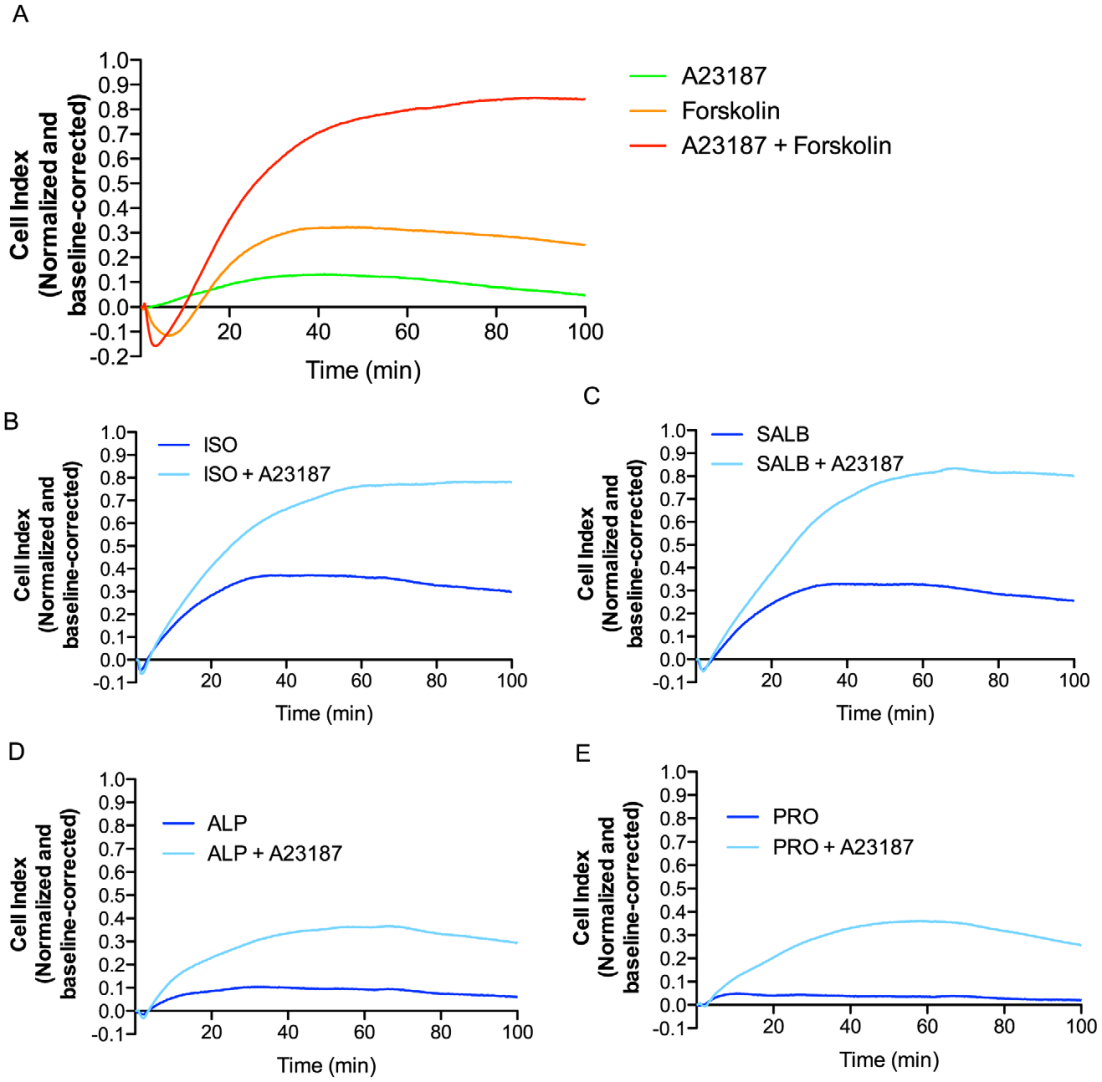
Increasing intracellular [Ca^2+^] accelerates the rapid ascending phase and maximum impedance response. (**A**) Impedance responses obtained following treatment with the Ca^2+^ ionophore A23187 (1 µM), the adenylyl cyclase activator forskolin (10 µM) or the combined stimulation with both. (**B–E**) Impedance responses obtained following stimulation with ISO (B), SALB (C), ALP (D) and PRO (E) (1 µM each) in the presence or absence of A23187 (1 µM). Data represent means of at least three independent experiments.

Interestingly, pre-treatment of cells with either 2-APB or BAPTA-AM significantly modulated the impedance responses not only for ligands in Groups I and II, but also Groups III and IV, which do not elicit a detectable increase in [Ca^2+^] (**Figure 6A,C–F**). Given that both 2-APB and BAPTA-AM not only inhibit ligand-induced Ca^2+^ mobilization, but also decrease the basal intracellular [Ca^2+^] (**Figure 6G**), we hypothesized that a minimal concentration of intracellular Ca^2+^ is required to initiate the cellular events underlying the ascending phases of the impedance response. To test this hypothesis, we pre-treated cells with the sarco/endoplasmic reticulum Ca^2+^ ATPase inhibitor thapsigargin (TG), which will lead to an increase in intracellular Ca^2+^ by preventing the reuptake of Ca^2+^, thus emptying the intracellular stores. Since the intracellular Ca^2+^ stores are already empty, ISO does not elicit an additional Ca^2+^ release following TG inhibition (**Figure S8A**). Yet, in contrast with what was observed with 2-APB and BAPTA-AM, and despite the absence of a *de novo* Ca^2+^ release, the TG treatment does not inhibit the rapid ascending phase of the impedance response (**Figure S8B**). In fact, it modestly potentiated the maximal response while slightly reducing the slope of the slow ascending phase. Taken together, these data support the notion that it is the intracellular [Ca^2+^] itself, more than the *de novo* Ca^2+^ release, that is the prime determinant of the ascending phases. The fundamental role of Ca^2+^ in the impedance response is further demonstrated by the effect of the Ca^2+^ ionophore A23187 on the impedance responses (**Figure 7**). On its own, the ionophore elicits only a weak positive impedance response. However, it greatly potentiates the impedance responses obtained upon stimulation with either β-adrenergic ligands or the direct activator of adenylyl cyclase, forskolin, yielding responses with faster kinetics of the ascending phase and a higher overall maximum response. This effect is particularly evident when considering the forskolin-stimulated impedance response, which consists of a long transient negative phase and a slow rise to a relatively modest maximum response in the absence of the Ca^2+^ionophore. Taken together, these data explicitly demonstrate the importance of Ca^2+^ in the impedance response and indicate that, under normal conditions, a minimal [Ca^2+^] is needed to yield an increase in the β_2_AR-promoted impedance response while additional Ca^2+^ mobilization by Group I and II ligands further accelerates the rapid ascending phase.

### Distinct impedance signatures detected in rat aortic vascular smooth muscle cells

**Figure 8:**
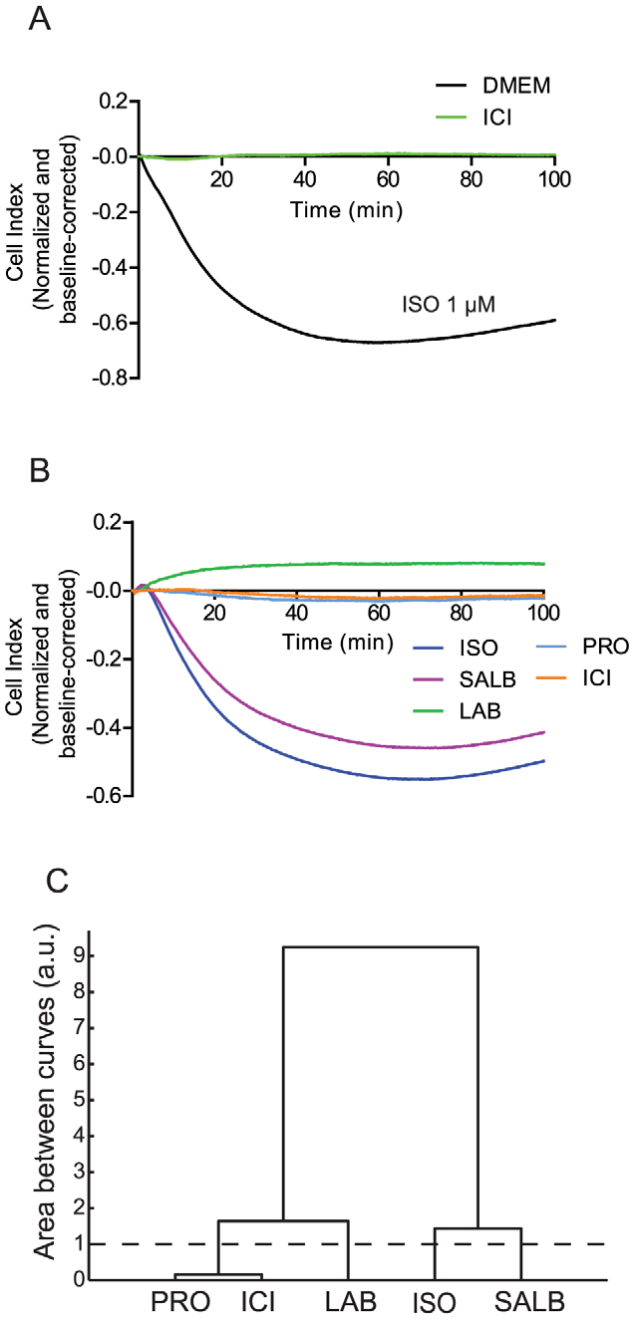
β-adrenergic ligand impedance responses in rat aortic vascular smooth muscle cells (VSMCs). (**A**) Pre-treatment with the β_2_-selective antagonist ICI118,551 (100 nM) for 1 hour completely abolishes the impedance response obtained following stimulation with 1 µM ISO in VSMCs. (**B**) Impedance signatures for β-adrenergic ligands representing each of the 5 compound classes defined in 6HisHA-β_2_AR-HEKS cells. (**C**) Complete linkage hierarchical clustering of ligand impedance responses determined by comparing the area between individual curves (see *Materials and Methods*). The dashed line represents the calculated threshold value defining compound dissimilarity. Data represent the means from at least three independent experiments.

To explore the applicability of impedance-based monitoring of the signaling activity of a GPCR in its native cellular context, we assessed the β_2_AR response in rat aortic vascular smooth muscle cells (VSMCs). As was the case in β_2_AR-expressing HEK293S cells, ISO induced a response that was completely abolished upon pre-treatment with the β_2_AR-selective antagonist ICI (**Figure 8A**). However, the shape of the impedance response was radically different in VSMCs, indicating that cellular response to receptor activation is cell type-specific. We next assessed the impedance responses induced upon treatment with ligands representing each of the 5 compound classes defined above (**Figure 8B**). Using the same clustering criteria as in the 6HisHA-β_2_AR-HEK293S cells, distinct impedance signatures for ISO (Group I), salbutamol (Group II) and labetalol (Group III) were observed. Propranolol (Group IV) and ICI (Group V) generated distinct signatures from these other compounds, but could not be distinguished from each other in VSMCs (**Figure 8C**), emphasizing the cell-type specificity of the response. Altogether, these data indicate that despite the fact that the magnitude and direction of the responses are cell-type specific, quantitative analysis of impedance responses can be used to detect distinct ligand signatures in primary cell cultures.

## Discussion

In the present study, we demonstrate that impedance responses provide an integrative assessment of the cellular consequence to GPCR stimulation, representing a holistic readout of the various signaling events elicited in real-time. In addition, clustering analyses of the impedance responses provided a means to sub-classify ligands in a manner that was predictive of their signaling profiles and revealed a richer signaling texture among β-adrenergic ligands than previously envisaged.

Analysis of the β_2_AR-promoted impedance response to ISO stimulation revealed the contribution of both G_s_ and G_i_ coupling, Gβγ-dependent signaling, as well as cAMP production, ERK1/2 activation and a novel Ca^2+^ mobilization response to the overall changes in cellular impedance. The observation that elements of the impedance response that were blocked upon G_s_ or G_i_ inactivation following chronic treatment with CTX and PTX, respectively, could not be fully recapitulated by acute pharmacological inhibition of cAMP or ERK1/2 pathways suggests the contribution of additional G protein-dependent signaling. However, we cannot rule out the possibility that chronic inhibition of G proteins leads to cellular changes (e.g. changes in expression of signaling or adhesion proteins) that indirectly affect the impedance response. Our analysis also provided evidence that both cAMP and ERK1/2 pathways converge on a common downstream effector that mediates their contribution to the impedance response, although the identity of this target remains unknown.

The impedance responses generated following β_2_AR stimulation was found to be extremely sensitive to inhibition of intracellular Ca^2+^ mobilization. Although the β_2_AR has previously been shown to increase intracellular [Ca^2+^] through modulation of L-type voltage-gated Ca^2+^ channels [29] and the ryanodine receptor [30], our data implicate a novel IP_3_-gated release of Ca^2+^ from intracellular stores. Ca^2+^ is a well-established regulator of actin cytoskeleton dynamics [31], [32] and plays an important role in controlling cellular adhesion and morphology, through effectors such as cadherins [33] and annexins [34], potentially providing a key mechanistic link between β_2_AR stimulation and the impedance changes observed. Although we found this *de novo* Ca^2+^ release to contribute to the rapid ascending phase of Groups I and II ligands, the impedance responses of ligands that do not themselves induce a Ca^2+^ mobilization are also sensitive to a disruption of intracellular Ca^2+^ homeostasis, stressing the importance of Ca^2+^ in the cellular mechanisms that initiate an impedance response. We propose that the normal resting concentration of intracellular Ca^2+^ is necessary and sufficient to permit the cytoskeleton to respond to subsequent signaling events, such as cAMP production or ERK1/2 activation, and that ligands that induce an additional increase in intracellular [Ca^2+^] further sensitize the cell to these signaling inputs leading to an enhancement of the impedance response. Thus, the overall impedance signature of a given ligand truly represents the integration of the signaling pathways activated.

The contribution of multiple signaling events to the overall impedance response challenges some of the efforts that have been made to reduce such integrated responses to the engagement of a single G protein [17], [18]. It is now widely accepted that many GPCRs promiscuously couple to and activate signaling through multiple G protein subtypes [3] as well as G protein-independent pathways. In the current study, we observed that both G_s_- and G_i_-dependent signaling were integrated in the overall β_2_AR-promoted response. Moreover, the fact that the signatures obtained in VSMCs were dramatically different from those observed in 6HisHA-β_2_AR-HEK293S cells highlights the cell-type dependency of the impedance response, which likely reflects differences in the relative abundance of signaling molecules and/or mediators underlying the changes in cell shape and adhesion that give rise to the impedance responses. These observations highlight the inherent complexity of signaling systems and their measurement and demonstrate the diversity of cellular responses that can be generated by the stimulation of a given GPCR in different cell contexts. The ability to detect and dissect these complex cellular responses, as demonstrated in the present study, provides information concerning the full signaling repertoire of a receptor that can be more useful in drug discovery than the attempt to ascribe a single signaling pathway to a receptor.

The fact that multiple signaling events are integrated in the impedance response makes it an appealing platform to classify compounds based on their signaling repertoire. Clustering of the impedance responses generated by a series of β-adrenergic ligands revealed an unprecedented level of ligand texture at the β_2_AR, demonstrating the existence of at least five groups of compounds with distinct impedance signatures. Previous work assessing the signaling profiles of β-adrenergic ligands towards cAMP and ERK1/2 pathways alone using endpoint-specific assays proposed the existence of 3 distinct compound classes [11]. The ability of impedance measurements to detect additional ligand diversity likely reflects the contribution of other pathways not previously assessed. Furthermore, differences in ligand signaling profiles between this study and the former demonstrate the critical influence of assay conditions on the reported signaling properties of a ligand. In the present study, cAMP accumulation and ERK1/2 activation were measured in conditions identical to those used for impedance measurements. Without serum starvation, ligands such as propranolol and ICI did not generate a detectable ERK1/2 response, as previously reported [11], [12]. However, differences in their respective impedance responses indicated distinctions in the overall cellular response induced by ICI and propranolol, which had not previously been observed.

Although cell-based, integrative assays have begun to be employed in some screening applications [35]–[38], our results represent a first proof-of-principle of how they can be used not only to identify potential ligands for a given target, but also to differentiate classes of compounds with specific signaling profiles that may correlate with therapeutic or adverse effects. We also demonstrate the unique ability of integrative assays such as impedance measurements to illuminate novel biology not yet revealed by traditional single endpoint assays. The approaches described should be universally applicable to all label-free platforms that provide an integrative cell response, including both impedance-based systems and those based on dynamic mass redistribution. Most certainly, analytical integrative methodologies such as the one presented here, will provide a more in-depth exploration of cellular signaling dynamics and should be transformative in the future of drug discovery.

## Materials and Methods

### Materials and reagents

Gallein was obtained from Tocris, U0126 from Cayman, and bucindolol was a generous gift from Dr. Michael Bristow (University of Colorado Health Sciences Center, Denver, CO). Cell culture reagents were from Wisent Incorporated. Other reagents were obtained from Sigma-Aldrich unless stated otherwise.

### Stable cell lines and transfection

The pIREShygro_6HisHA-β_2_AR construct was generated as follows: The 6HisHA-β_2_AR gene was amplified by PCR from the previously described vector pCDNA3RSV-6HisHA-β_2_AR[39] using sense and antisense primers containing *BsrGI* and *NheI* restriction sites, respectively. The amplified 6HisHA-β_2_AR sequence was digested with *BsrGI/NheI* and then subcloned in a *BsrGI/NheI*-digested pIREShygro3 vector (Clontech, Mountain View, CA). The 6HisHA-β_2_AR-HEK392S polyclonal cell line was generated by transfecting the pIREShygro_6HisHA-β_2_AR construct in HEK293S cells [40] and selecting vector-inserted cells by growth in culture media containing 100 µg/ml hygromycin B (WISENT inc, St-Bruno, Quebec, CAN). The 6HisHA-β_2_AR-HEK293S stable cell line was found to express 1.07±0.14 pmol of 6HisHA-β_2_AR per mg of membrane protein as determined by a ^125^I-CYP radioligand binding assay. Cells were routinely grown at 37°C with 5% CO_2_ in Dulbecco's modified Eagle's medium supplemented with 5% fetal bovine serum. For Ca^2+^ assays and impedance assays in the presence of the MEK dominant negative mutant, 6HisHA-β_2_AR HEK293S cells were transiently transfected with the obelin biosensor [41] or the MEK1-K97A-Flag mutant [42], respectively, using polyethylenimine and assayed 48 hours later.

### Cellular impedance assay

The xCELLigence system (Roche Applied Science) was employed to measure changes in cellular impedance following ligand stimulation [43]. Briefly, this assay is based on the principle that the adhesion of cultured cells directly onto an array of equally-distributed microelectrodes embedded at the bottom of wells of a microtitre plate induces changes in the local ionic environment at the electrode/solution interface, conferring an increase in electrode impedance. As a result, any changes in cell physiological properties that modulate the physical contact between cell and electrode, such as changes in morphology and/or adhesion, will be reflected by changes in the measured impedance, as defined by the cell index variable. A measurement is made in the presence of growth medium prior to cell seeding to determine the background cell index in each well of a 96-well E-plate (Roche Applied Science), which is subtracted from the cell index values generated following cell attachment. Cells were plated at a cell density of 20 000 cells/well and grown for 16–20 hours before ligand addition in the RTCA SP device station (Roche Applied Science). When cells were treated with cholera toxin or pertussis toxin, they were plated at a cell density of 10 000 cells/well, treated with toxins after 24 hours and stimulated with ligand 40 hours after seeding. Experimental variation in cell index values at the time of treatment was limited to ±20%, which considerably increased the reproducibility of the data obtained. Cells were treated with compounds as indicated and cell index values were obtained immediately following ligand stimulation every 20 seconds for a total time of at least 100 minutes. Cell index values were normalized by dividing by the cell index at the time of ligand addition and baseline-corrected by subtracting the cell index obtained in vehicle-treated conditions (see **Figure S9**). All data presented in figures represent means of at least three independent experiments.

### cAMP accumulation assay

Cells were plated, grown in 96-well plates and treated with ligand as described for the cellular impedance assay. cAMP accumulation was measured using the HTFR-cAMP dynamic kit (Cisbio). After 10 minutes of ligand stimulation, cells were lysed on ice in the lysis buffer provided by the manufacturer and immediately snap-froze at −80°C for at least 4 hours. After thawing on ice, 10 µl per well of lysate was transferred to a 384-well plate (Lumitrac 200, Greiner Bio-one), and cAMP measurements were performed as per manufacturer's instructions.

### ERK1/2 activation assay

Cells were plated, grown in 96-well plates and treated with ligand as described for the cellular impedance assay. ERK1/2 activation was measured using the ERK1/2 AlphaScreen Surefire kit (Perkin Elmer). Cells were lysed on ice after 2 minutes of ligand stimulation in the lysis buffer provided by the manufacturer and placed immediately at −20°C overnight. After thawing on ice, 5 µl were transferred to 384-well ProxiPlates (Perkin Elmer) and ERK1/2 activation was measured as per manufacturer's instructions.

### Obelin Ca^2+^ biosensor assay

Cells were plated, grown in 96-well microtitre plates and transfected as described above. Forty-eight hours after transfection, growth medium was replaced with Tyrode's solution (1 mM CaCl_2_, 140 mM NaCl, 2.7 mM KCl, 900 µM MgCl_2_, 37 µM NaH_2_PO_4_, 5.6 mM d-glucose, 8.3 mM NaHCO_3_ and 12.6 mM Hepes, pH 7.5) and cells were incubated with 5 µM coelanterazine H (Nanolight Technology, Pinetop, AZ, USA) for 2 hours. Intracellular Ca^2+^ concentration is reflected by the magnitude of bioluminescence emitted by the obelin biosensor and data are expressed as relative luminescence units (RLU). Ligands were injected as indicated and bioluminescence was measured for 1 minute per condition using the SpectraMax L Microplate reader (Molecular Devices). Baseline readings were obtained for 5 seconds prior to ligand addition and subtracted from post-injection readings to obtain a ligand-induced Ca^2+^ response. Peak Ca^2+^ responses represent the maximum response in the largest peak obtained following ligand addition. Peaks were defined to have a minimum width of ten adjacent points.

### Data analysis

Data analysis was performed using Microsoft Excel (Microsoft, Redmond, WA, USA), GraphPad Prism 5 (GraphPad Software, La Jolla, CA, USA) or MATLAB (The MathWorks, Natick, MA, USA). To quantify the slope of the rapid ascending phase, a line was drawn from two data points after the minimum (to avoid artifacts from shallow minima) to maximum response, and the impedance response was bisected at the point farthest from that line. Then, a second line was drawn from two data points after minimum response to the first bisection point, and the impedance response was bisected again. Slope was calculated as the ratio between the difference in impedance and the difference in time between two data points after the minimum response and the second bisection point.

Differences in ligand impedance signatures were determined by calculating the area between their impedance curves. The area *A* between two curves *i* and *j* was estimated as
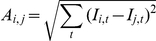
where *I_i,t_* is the impedance response of curve *i* at time *t*.

Clustering of the data was assessed using the visual assessment of tendency method (VAT) [44]. Briefly, arrays of the pairwise dissimilarities were sorted by placing the two ligands with the highest pairwise dissimilarity in the first and last rows, respectively. The array was then populated with the remaining ligands by minimizing the dissimilarity among adjacent compounds and maintaining the same order of compounds in rows and columns. To emphasize cluster structure among similar data points, the square root of the sorted dissimilarity maps were displayed.

For complete linkage hierarchical clustering, clustering trees were cut at 0.8 times the median of all pairwise dissimilarities.

## Supporting Information

Figure S1 **ISO-promoted impedance response is β_2_AR-specific.** (**A**) Impedance response upon ISO stimulation following 1 hour pre-treatment with the β_2_AR-selective antagonist ICI118,551 (ICI) or the β_1_AR-selective antagonist CGP20712A (10 µM each). (**B**) Concentration-response curves of ISO-promoted maximum impedance response following pre-treatment with increasing concentrations of ICI118,551. Data represent the means from three independent experiments (+/− SEM for B).
(TIF)

Figure S2 **Signaling and impedance responses in parental HEK293S and 6HisHA-β_2_AR-HEK293S cells.** (**A**) Comparison of the impedance responses observed in parental HEK293S and 6HisHA-β_2_AR-HEK293S stable cell lines following treatment with ISO (1 µM). (**B**) Impedance responses upon ISO stimulation in the presence or absence of a 1 hour pre-treatment with the β_2_AR-selective antagonist ICI118,551 (ICI, 10 µM) in the parental HEK293S cells, demonstrating that the response observed in these cells resulted from activation of endogenous β_2_AR. (**C,D**) The ISO-promoted cAMP production (C) and ERK1/2 activation (D) observed in the parental HEK293S expressing low levels of endogenous β_2_AR was potentiated by the overexpression of human β_2_AR in 6HisHA-β_2_AR-HEK293S cells. Accumulation of cAMP was detected using the EPAC cAMP biosensor (M. Leduc, B. Breton and N. Heveker, *et al. J. Pharmacol. Exp. Ther.*, 331 (2009), pp. 297–307). Data represent means of at least three independent experiments. Statistical significance of the differences between the conditions for panels C and D were assessed using Student's paired *t*-test. ** P<0.01, * P<0.05.
(TIF)

Figure S3 **Involvement of the MEK/ERK1/2 pathway in the ISO-promoted impedance response.** Cells were transfected or not (Mock) with the MEK1-K97A-Flag dominant negative mutant 48 hours prior the impedance measurements and treated with ISO (1 µM). Data represent means of three independent experiments.
(TIF)

Figure S4 **Comparison of the direct inhibition of Gα_s_, Gβ_i_ and Gβγ vs inhibition of cAMP and ERK1/2 pathways on the impedance response.** (**A**) Comparison of cholera toxin (CTX) and SQ22536 pre-treatments on the ISO-promoted impedance response (Figure 2A and 3A). (**B**) Comparison of pertussis toxin (PTX) and U0126 pre-treatments on the ISO-promoted impedance response (Figure 2B and 3A). (**C**) Comparison of gallein (Gall) and U0126 pre-treatments on the ISO-promoted impedance response (Figure 2B and 3D).
(TIF)

Figure S5 **Statistical analysis of the variability of impedance responses.** (**A–E**) Repeated impedance responses were obtained for ligands from each of the five groups of compounds. In each case, 3–7 independent measurements were made and mean impedance values (CI) were determined for each time-point. The mean impedance responses are shown in solid, colored lines whereas the dotted black lines represent the standard error of the mean (SEM) for each time-point.
(TIF)

Figure S6 **Visual assessment of clustering tendency of -adrenergic ligand impedance signatures.** Gray levels represent the extent of dissimilarity between ligands as determined by area between the curves, ranging from black (smallest difference) to white (largest difference). Ligands are ordered based on similarities in the area between their curves. Ligands with similar impedance signatures (small differences in area between the curves) are visualized as dark clusters along the diagonal. See *Materials and Methods* for additional information.
(TIF)

Figure S7 **ISO-induced Ca^2+^ response is β_2_AR-specific.** 6HisHA-β_2_AR-HEK293S were pre-treated or not with the β_2_AR-selective antagonist ICI118,551 (100 nM) for 1 hour before stimulation with 1 µM ISO. Data represent means of three independent experiments (± SEM).
(TIF)

Figure S8 **Effect of thapsigargin on ISO-induced Ca^2+^ and impedance responses.** ISO-induced Ca^2+^ response (**A**) and impedance response (**B**) upon pre-treatment or not with thapsigargin (TG, 5 µM) for 30 minutes. Data represent means (+/− SEM for **A**) from at least three independent experiments.
(TIF)

Figure S9 **Normalization and baseline-correction of impedance responses.** (1) A measurement of cell index is made in the presence of growth medium prior to cell seeding to determine the background cell index in each well, which is subtracted from the cell index values generated by cell attachment. Cells are grown for 16–20 hours before ligand treatment. (2) Cell index values are obtained immediately following ligand treatment every 20 seconds for a total time of at least 100 minutes. (3) Cell index values are normalized by dividing by the cell index at the time of ligand addition and (4) baseline-corrected by subtracting the cell index obtained in vehicle-treated conditions. See *Materials and Methods* for more details.
(TIF)

## References

[1] Lagerström MC, Schiöth HB (2008). Structural diversity of G protein-coupled receptors and significance for drug discovery.. Nature reviews Drug discovery.

[2] Imming P, Sinning C, Meyer A (2006). Drugs, their targets and the nature and number of drug targets.. Nature reviews Drug discovery.

[3] Hermans E (2003). Biochemical and pharmacological control of the multiplicity of coupling at G-protein-coupled receptors.. Pharmacology & Therapeutics.

[4] Bockaert J, Fagni L, Dumuis A, Marin P (2004). GPCR interacting proteins (GIP).. Pharmacology & therapeutics.

[5] Kenakin T (2010). Functional Selectivity and Biased Receptor Signaling.. The Journal of pharmacology and experimental therapeutics.

[6] Stallaert W, Christopoulos A, Bouvier M (2011). Ligand functional selectivity and quantitative pharmacology at G protein-coupled receptors.. Expert Opinion on Drug Discovery.

[7] Galandrin S, Oligny-Longpré G, Bouvier M (2007). The evasive nature of drug efficacy: implications for drug discovery.. Trends in pharmacological sciences.

[8] Kenakin T, Miller LJ (2010). Seven Transmembrane Receptors as Shapeshifting Proteins: The Impact of Allosteric Modulation and Functional Selectivity on New Drug Discovery.. Pharmacological Reviews.

[9] Violin JD, Dewire SM, Yamashita D, Rominger DH, Nguyen L (2010). Selectively Engaging beta-Arrestins at the Angiotensin II Type 1 Receptor Reduces Blood Pressure and Increases Cardiac.. Journal of Pharmacology and Experimental Therapeutics.

[10] Wei H, Ahn S, Shenoy SK, Karnik SS, Hunyady L (2003). Independent beta-arrestin 2 and G protein-mediated pathways for angiotensin II activation of extracellular signal-regulated kinases 1 and 2.. Proceedings of the National Academy of Sciences of the United States of America.

[11] Galandrin S, Bouvier M (2006). Distinct signaling profiles of beta1 and beta2 adrenergic receptor ligands toward adenylyl cyclase and mitogen-activated protein kinase reveals the pluridimensionality of efficacy.. Mol Pharmacol.

[12] Azzi M, Charest PG, Angers S, Rousseau G, Kohout T (2003). Beta-arrestin-mediated activation of MAPK by inverse agonists reveals distinct active conformations for G protein-coupled receptors.. Proceedings of the National Academy of Sciences of the United States of America.

[13] Noma T, Lemaire A, Prasad SVN, Barki-Harrington L, Tilley DG (2007). beta-arrestin-mediated beta1-adrenergic receptor transactivation of the EGFR confers cardioprotection.. Journal of Clinical Investigation.

[14] Masri B, Salahpour A, Didriksen M, Ghisi V, Beaulieu J-M (2008). Antagonism of dopamine D2 receptor/beta-arrestin 2 interaction is a common property of clinically effective antipsychotics.. Proceedings of the National Academy of Sciences of the United States of America.

[15] Walters RW, Shukla AK, Kovacs JJ, Violin JD, Dewire SM (2009). Beta-Arrestin1 mediates nicotinic acid – induced flushing , but not its antilipolytic effect, in mice.. The Journal of Clinial Investigation.

[16] Yu N, Atienza JM, Bernard J, Blanc S, Zhu J (2006). Real-time monitoring of morphological changes in living cells by electronic cell sensor arrays: an approach to study G protein-coupled receptors.. Analytical chemistry.

[17] Peters MF, Vaillancourt F, Heroux M, Valiquette M, Scott CW (2010). Comparing label-free biosensors for pharmacological screening with cell-based functional assays.. Assay Drug Dev Technol.

[18] Peters MF, Scott CW (2009). Evaluating Cellular Impedance Assays for Detection of GPCR Pleiotropic Signaling and Functional Selectivity.. Journal of Biomolecular Screening.

[19] Scandroglio P, Brusa R, Lozza G, Mancini I, Petro R (2010). Evaluation of Cannabinoid Receptor 2 and Metabotropic Glutamate Receptor 1 Functional Responses Using a Cell Impedance-Based Technology.. J Biomol Screen.

[20] Schröder R, Janssen N, Schmidt J, Kebig A, Merten N (2010). Deconvolution of complex G protein-coupled receptor signaling in live cells using dynamic mass redistribution measurements.. Nature biotechnology.

[21] Abassi YA, Xi B, Zhang W, Ye P, Kirstein SL (2009). Kinetic cell-based morphological screening: prediction of mechanism of compound action and off-target effects.. Chemistry & biology.

[22] Daaka Y, Luttrell LM, Lefkowitz RJ (1997). Switching of the coupling of the beta2-adrenergic receptor to different G proteins by protein kinase A.. Nature.

[23] Levis MJ, Bourne HR (1992). Activation of the alpha subunit of Gs in intact cells alters its abundance, rate of degradation, and membrane avidity.. The Journal of cell biology.

[24] Lehmann DM, Seneviratne AMPB, Smrcka AV (2008). Small molecule disruption of G protein beta gamma subunit signaling inhibits neutrophil chemotaxis and inflammation.. Molecular pharmacology.

[25] Hoffmann C, Leitz MR, Oberdorf-Maass S, Lohse MJ, Klotz K-N (2004). Comparative pharmacology of human beta-adrenergic receptor subtypes–characterization of stably transfected receptors in CHO cells.. Naunyn-Schmiedeberg's archives of pharmacology.

[26] Baker JG (2005). The selectivity of beta-adrenoceptor antagonists at the human beta1, beta2 and beta3 adrenoceptors.. British journal of pharmacology.

[27] Hershberger RE, Wynn JR, Sundberg L, Bristow MR (1990). Mechanism of action of bucindolol in human ventricular myocardium.. Journal of cardiovascular pharmacology.

[28] Jain AK, Murty MN, Flynn PJ (1999). Data clustering: a review.. ACM Computing Surveys.

[29] Benitah J-P, Alvarez JL, Gómez AM (2010). L-type Ca(2+) current in ventricular cardiomyocytes.. Journal of molecular and cellular cardiology.

[30] Kushnir A, Marks AR (2010). The ryanodine receptor in cardiac physiology and disease.. Adv Pharmacol.

[31] Marston S (1995). Ca2+-dependent Protein Switches in Actomyosin Based Contractile Systems.. International Journal of Biochemistry & Cellular Biology.

[32] Janmey P (1994). Phosphoinositides and calcium as regulators of cellular actin assembly and disassembly.. Annual Review of Physiology.

[33] van Roy F, Berx G (2008). The cell-cell adhesion molecule E-cadherin.. Cellular and molecular life sciences : CMLS.

[34] Gerke V, Creutz CE, Moss SE (2005). Annexins: linking Ca2+ signalling to membrane dynamics.. Nature reviews Molecular cell biology.

[35] Fang Y, Ferrie AM (2008). Label-free optical biosensor for ligand-directed functional selectivity acting on beta(2) adrenoceptor in living cells.. FEBS letters.

[36] Dodgson K, Gedge L, Murray DC, Coldwell M (2009). A 100K well screen for a muscarinic receptor using the Epic label-free system–a reflection on the benefits of the label-free approach to screening seven-transmembrane receptors.. Journal of receptor and signal transduction research.

[37] Atienza JM, Yu N, Wang X, Xu X, Abassi Y (2006). Label-free and real-time cell-based kinase assay for screening selective and potent receptor tyrosine kinase inhibitors using microelectronic sensor array.. Journal of biomolecular screening : the official journal of the Society for Biomolecular Screening.

[38] McGuinness RP, Proctor JM, Gallant DL, van Staden CJ, Ly JT (2009). Enhanced selectivity screening of GPCR ligands using a label-free cell based assay technology.. Combinatorial chemistry & high throughput screening.

[39] Lavoie C, Mercier J-F, Salahpour A, Umapathy D, Breit A (2002). Beta 1/beta 2-adrenergic receptor heterodimerization regulates beta 2-adrenergic receptor internalization and ERK signaling efficacy.. The Journal of biological chemistry.

[40] Reeves PJ, Thurmond RL, Khorana HG (1996). Structure and function in rhodopsin: high level expression of a synthetic bovine opsin gene and its mutants in stable mammalian cell lines.. Proceedings of the National Academy of Sciences of the United States of America.

[41] Markova SV, Vysotski ES, Blinks JR, Burakova LP, Wang B-C (2002). Obelin from the bioluminescent marine hydroid Obelia geniculata: cloning, expression, and comparison of some properties with those of other Ca2+-regulated photoproteins.. Biochemistry.

[42] Scott MGH, Pierotti V, Storez H, Lindberg E, Thuret A (2006). Cooperative regulation of extracellular signal-regulated kinase activation and cell shape change by filamin A and beta-arrestins.. Molecular and cellular biology.

[43] Solly K, Wang X, Xu X, Strulovici B, Zheng W (2004). Application of real-time cell electronic sensing (RT-CES) technology to cell-based assays.. Assay and drug development technologies.

[44] Bezdek JC, Hathaway RJ (2002). VAT: A tool for visual assessment of (cluster) tendency.. Proceedings of the international joint conference on neural networks.

