# Supplementary figures and images for: Impedance Responses Reveal β2-Adrenergic Receptor Signaling Pluridimensionality and Allow Classification of Ligands with Distinct Signaling Profiles
Source: PLoS One. 2012 Jan 5;7(1):e29420. doi: 10.1371/journal.pone.0029420 (PMC3252315; doi:10.1371/journal.pone.0029420)

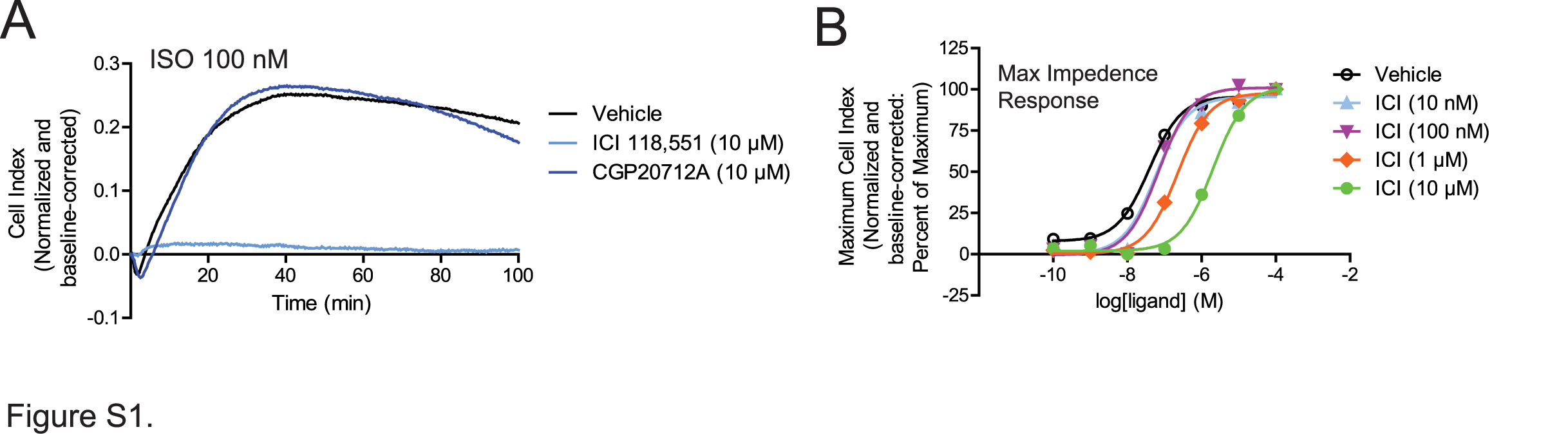

Supplement: Figure S1 — ISO-promoted impedance response is β2AR-specific. (A) Impedance response upon ISO stimulation following 1 hour pre-treatment with the β2AR-selective antagonist ICI118,551 (ICI) or the β1AR-selective antagonist CGP20712A (10 µM each). (B) Concentration-response curves of ISO-promoted maximum impedance response following pre-treatment with increasing concentrations of ICI118,551. Data represent the means from three independent experiments (+/− SEM for B). (TIF) [file pone.0029420.s001.tif]

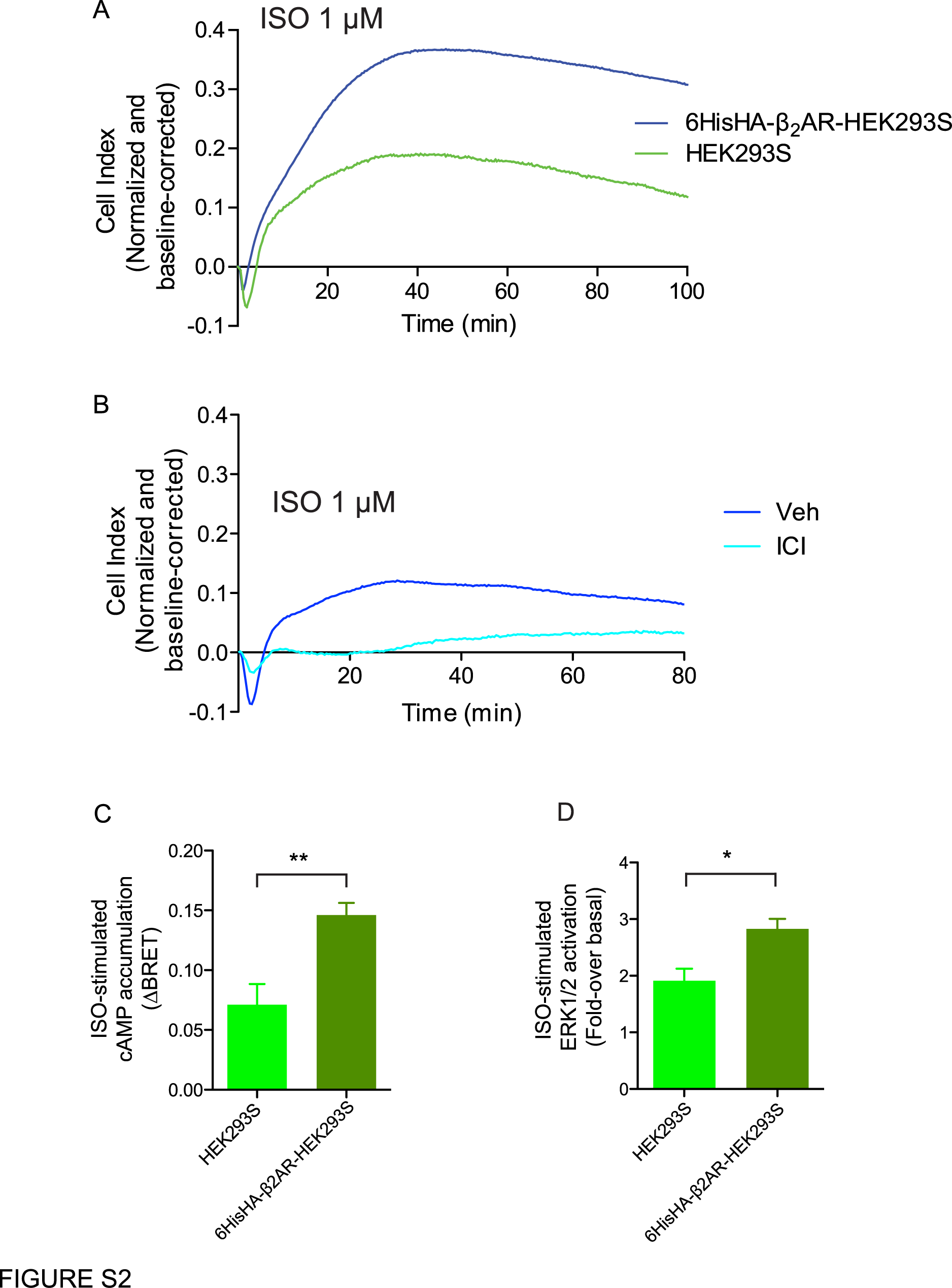

Supplement: Figure S2 — Signaling and impedance responses in parental HEK293S and 6HisHA-β2AR-HEK293S cells. (A) Comparison of the impedance responses observed in parental HEK293S and 6HisHA-β2AR-HEK293S stable cell lines following treatment with ISO (1 µM). (B) Impedance responses upon ISO stimulation in the presence or absence of a 1 hour pre-treatment with the β2AR-selective antagonist ICI118,551 (ICI, 10 µM) in the parental HEK293S cells, demonstrating that the response observed in these cells resulted from activation of endogenous β2AR. (C,D) The ISO-promoted cAMP production (C) and ERK1/2 activation (D) observed in the parental HEK293S expressing low levels of endogenous β2AR was potentiated by the overexpression of human β2AR in 6HisHA-β2AR-HEK293S cells. Accumulation of cAMP was detected using the EPAC cAMP biosensor (M. Leduc, B. Breton and N. Heveker, et al. J. Pharmacol. Exp. Ther., 331 (2009), pp. 297–307). Data represent means of at least three independent experiments. Statistical significance of the differences between the conditions for panels C and D were assessed using Student's paired t-test. ** P<0.01, * P<0.05. (TIF) [file pone.0029420.s002.tif]

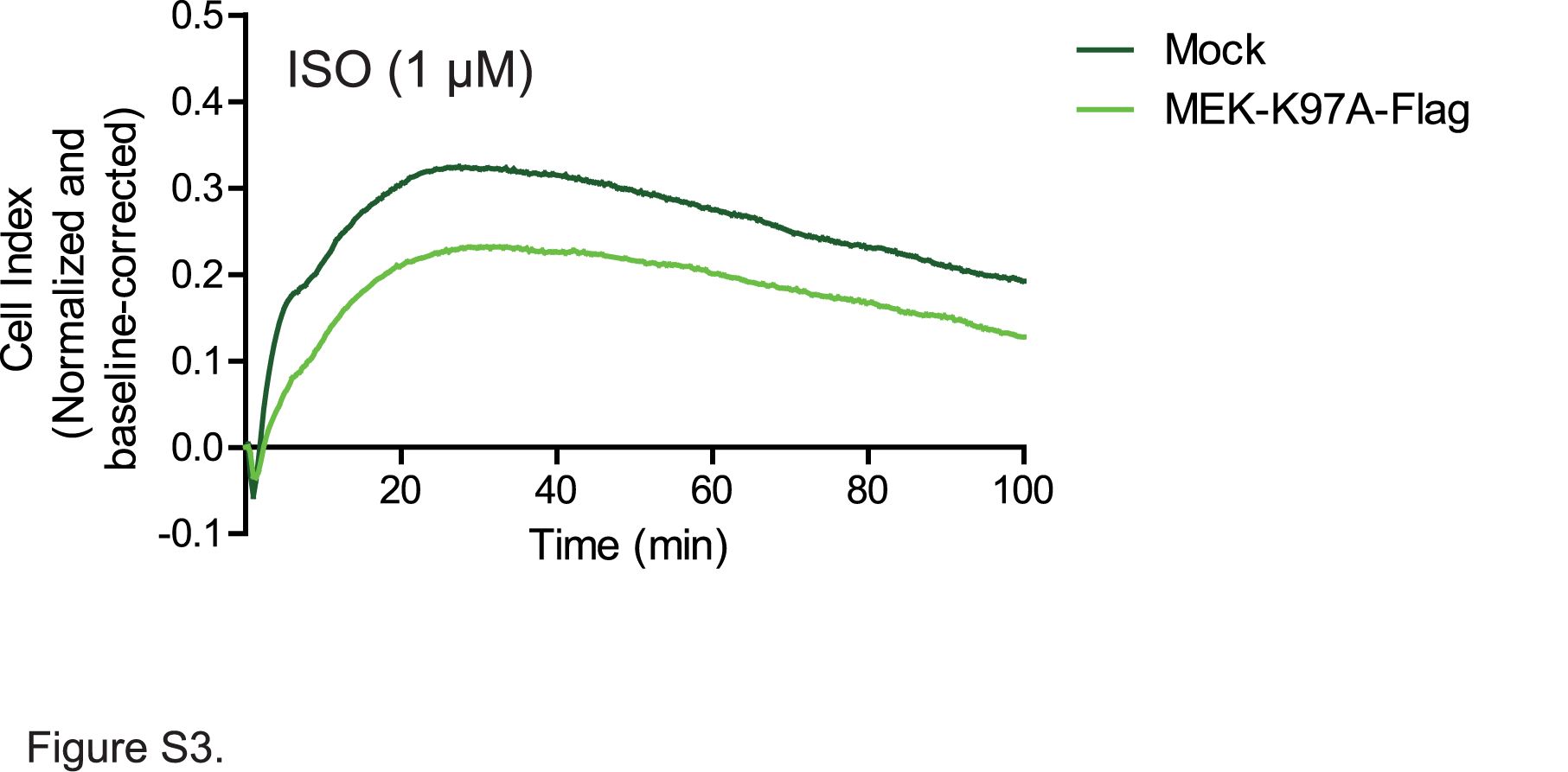

Supplement: Figure S3 — Involvement of the MEK/ERK1/2 pathway in the ISO-promoted impedance response. Cells were transfected or not (Mock) with the MEK1-K97A-Flag dominant negative mutant 48 hours prior the impedance measurements and treated with ISO (1 µM). Data represent means of three independent experiments. (TIF) [file pone.0029420.s003.tif]

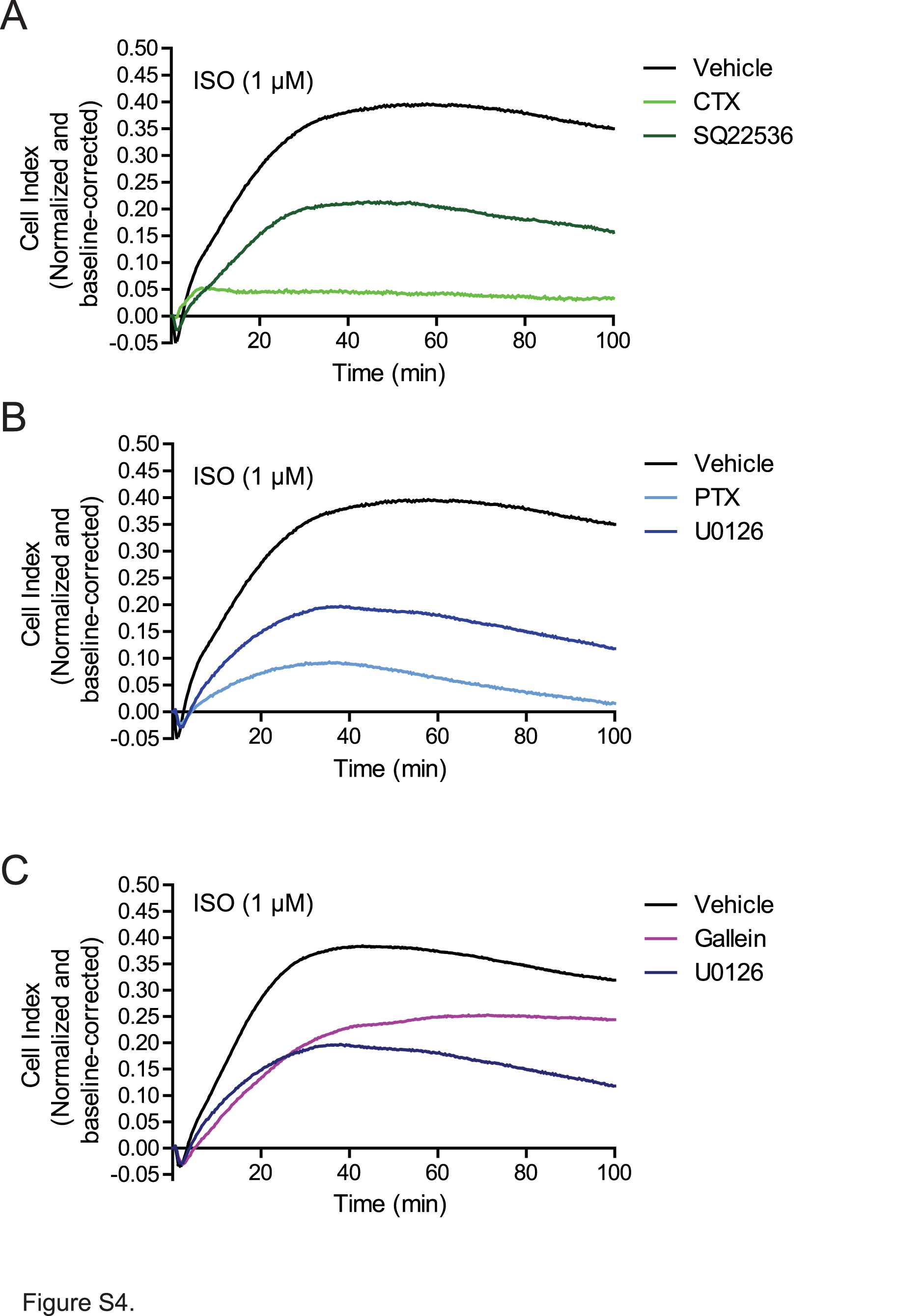

Supplement: Figure S4 — Comparison of the direct inhibition of Gαs, Gβi and Gβγ vs inhibition of cAMP and ERK1/2 pathways on the impedance response. (A) Comparison of cholera toxin (CTX) and SQ22536 pre-treatments on the ISO-promoted impedance response (Figure 2A and 3A). (B) Comparison of pertussis toxin (PTX) and U0126 pre-treatments on the ISO-promoted impedance response (Figure 2B and 3A). (C) Comparison of gallein (Gall) and U0126 pre-treatments on the ISO-promoted impedance response (Figure 2B and 3D). (TIF) [file pone.0029420.s004.tif]

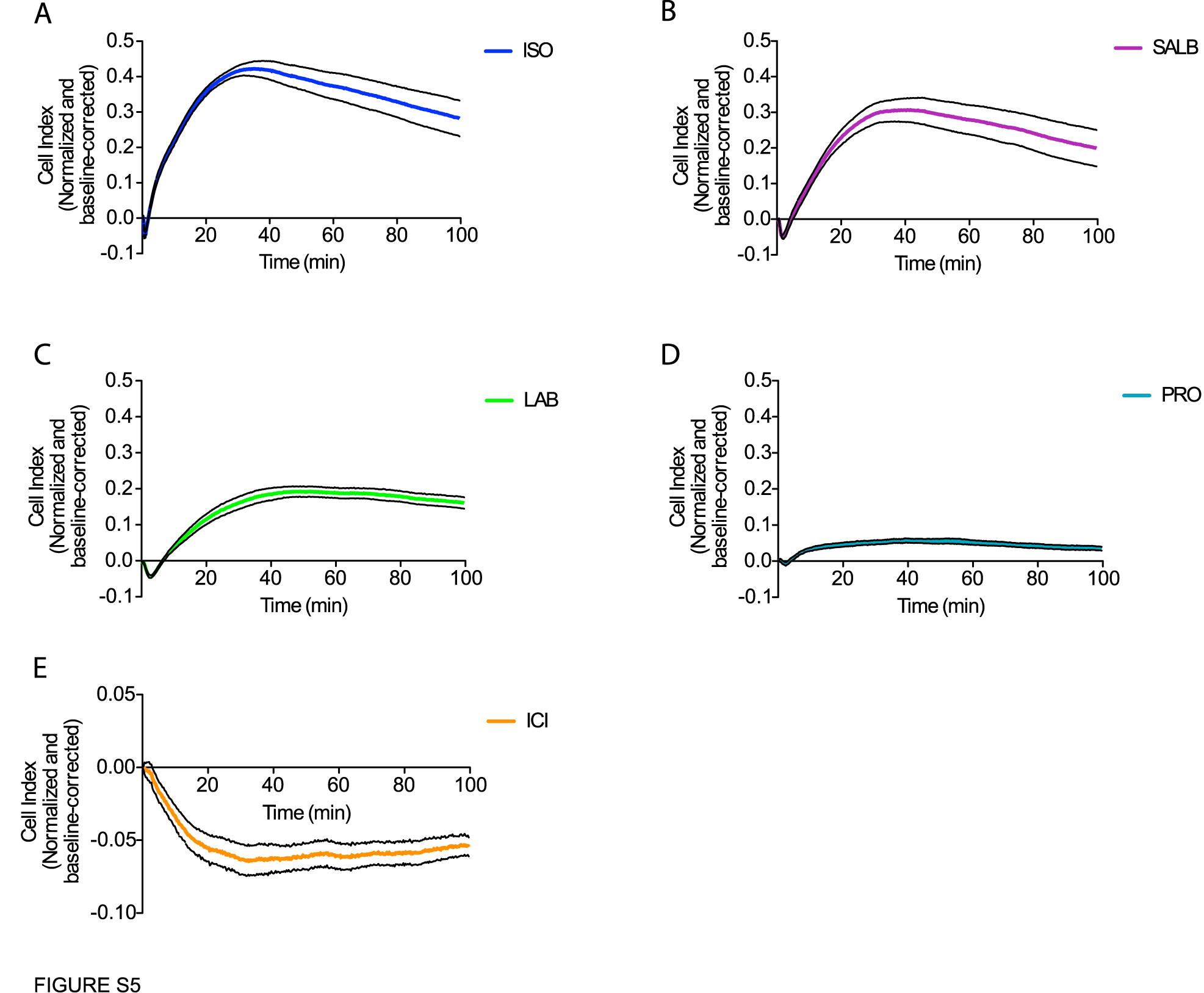

Supplement: Figure S5 — Statistical analysis of the variability of impedance responses. (A–E) Repeated impedance responses were obtained for ligands from each of the five groups of compounds. In each case, 3–7 independent measurements were made and mean impedance values (CI) were determined for each time-point. The mean impedance responses are shown in solid, colored lines whereas the dotted black lines represent the standard error of the mean (SEM) for each time-point. (TIF) [file pone.0029420.s005.tif]

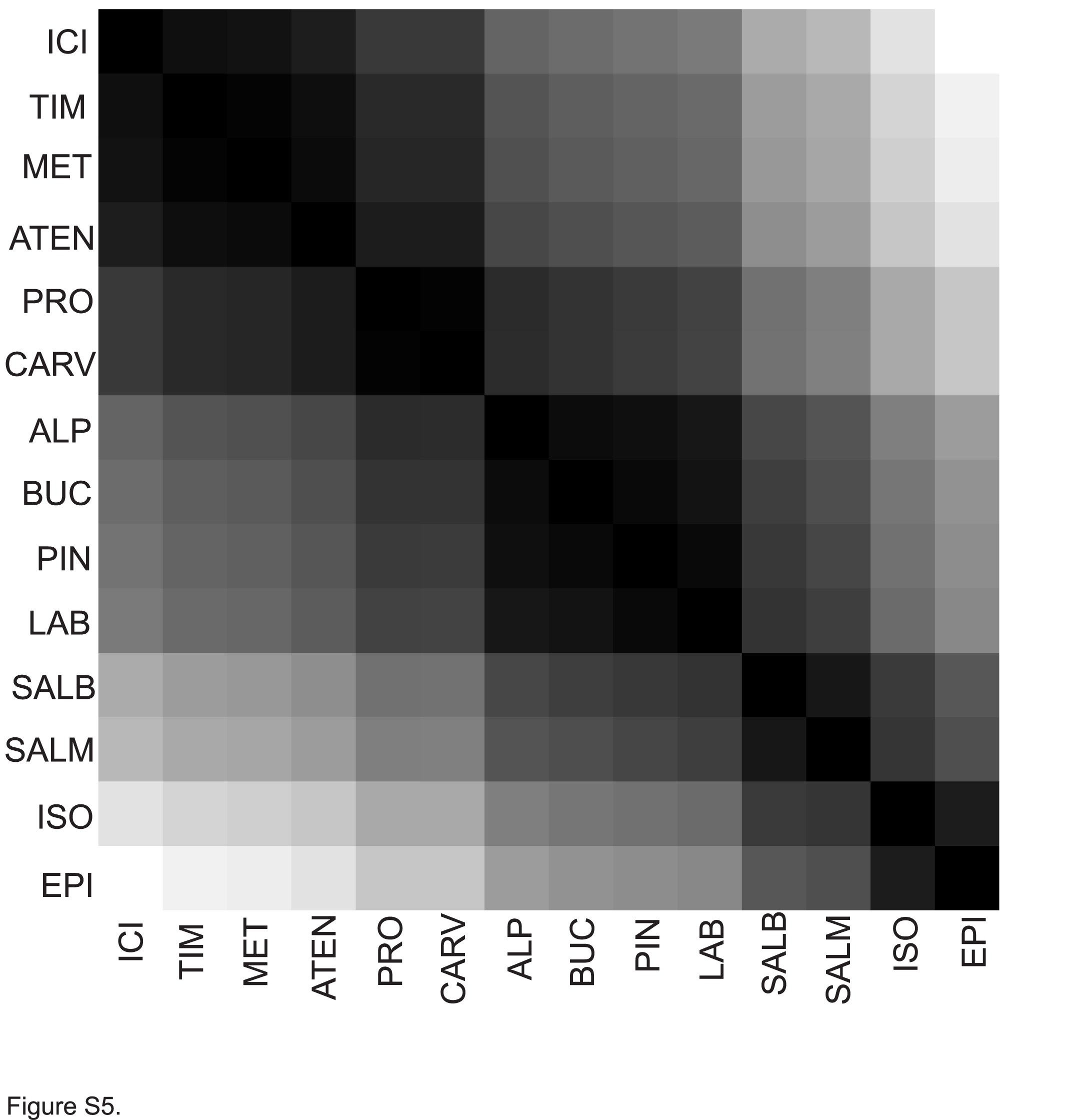

Supplement: Figure S6 — Visual assessment of clustering tendency of -adrenergic ligand impedance signatures. Gray levels represent the extent of dissimilarity between ligands as determined by area between the curves, ranging from black (smallest difference) to white (largest difference). Ligands are ordered based on similarities in the area between their curves. Ligands with similar impedance signatures (small differences in area between the curves) are visualized as dark clusters along the diagonal. See Materials and Methods for additional information. (TIF) [file pone.0029420.s006.tif]

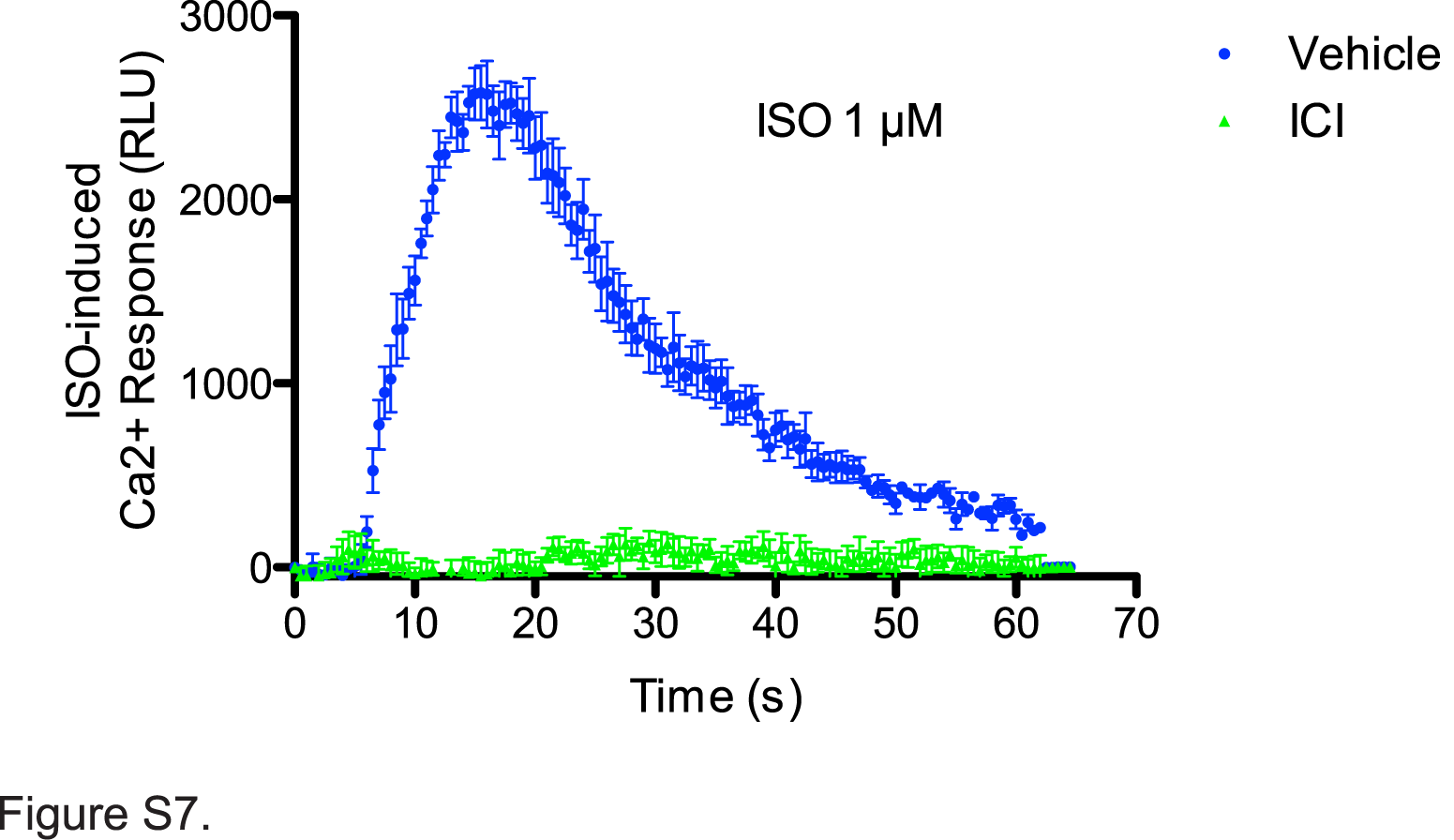

Supplement: Figure S7 — ISO-induced Ca2+ response is β2AR-specific. 6HisHA-β2AR-HEK293S were pre-treated or not with the β2AR-selective antagonist ICI118,551 (100 nM) for 1 hour before stimulation with 1 µM ISO. Data represent means of three independent experiments (± SEM). (TIF) [file pone.0029420.s007.tif]

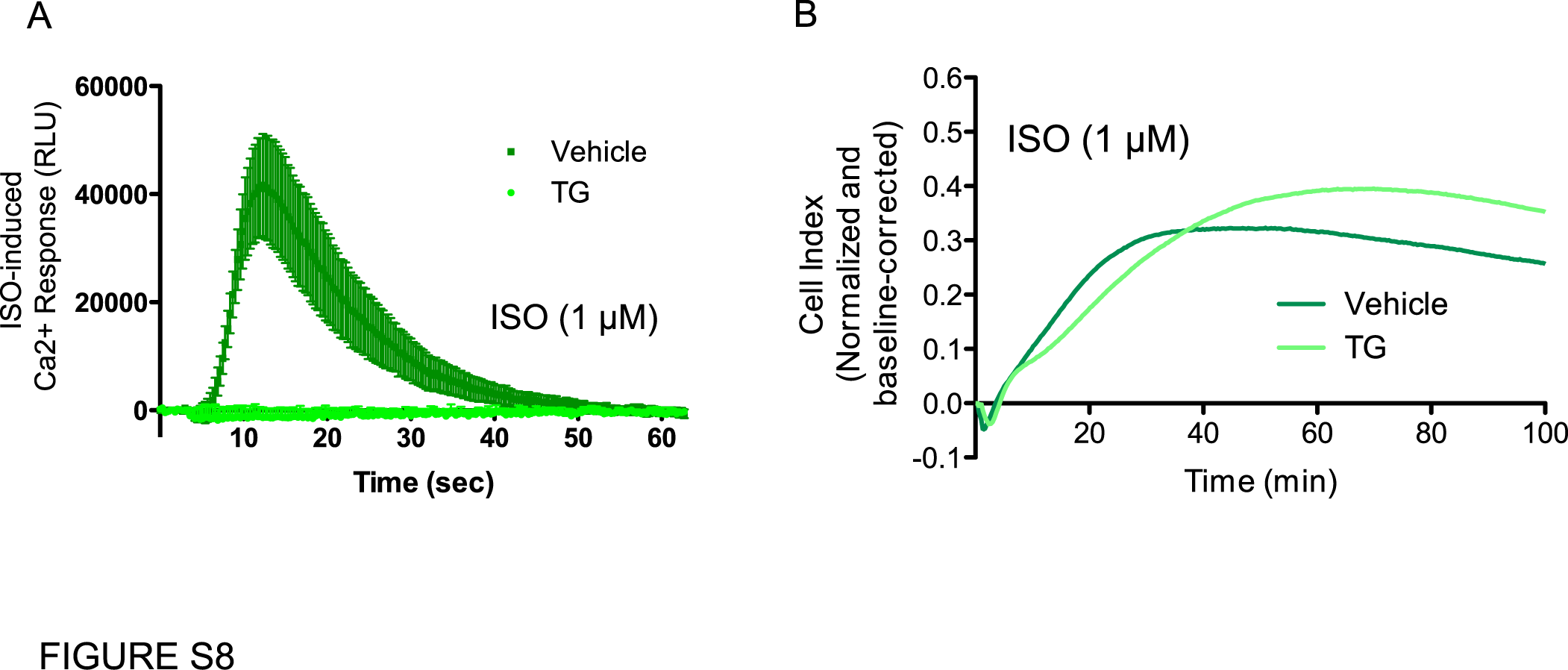

Supplement: Figure S8 — Effect of thapsigargin on ISO-induced Ca2+ and impedance responses. ISO-induced Ca2+ response (A) and impedance response (B) upon pre-treatment or not with thapsigargin (TG, 5 µM) for 30 minutes. Data represent means (+/− SEM for A) from at least three independent experiments. (TIF) [file pone.0029420.s008.tif]

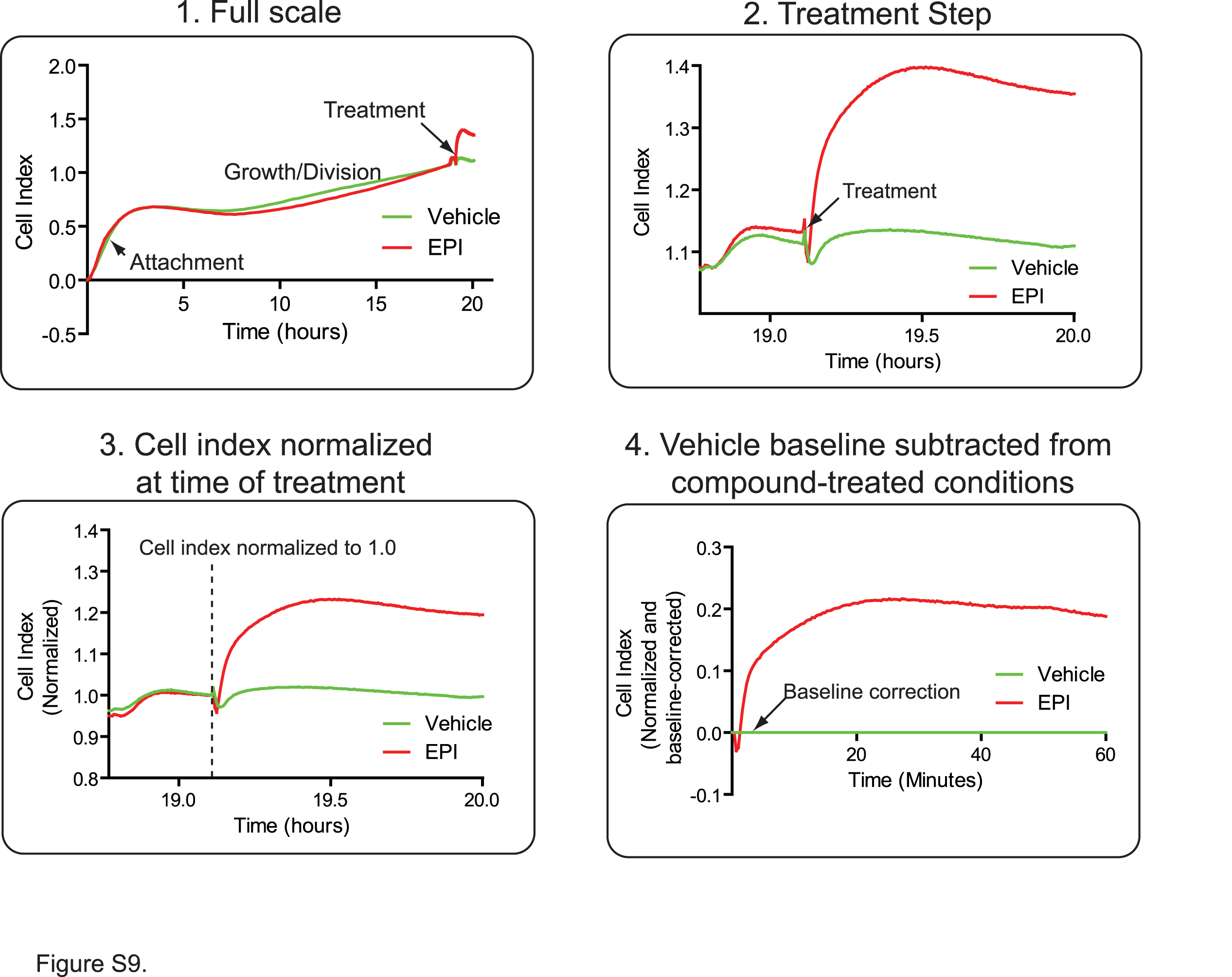

Supplement: Figure S9 — Normalization and baseline-correction of impedance responses. (1) A measurement of cell index is made in the presence of growth medium prior to cell seeding to determine the background cell index in each well, which is subtracted from the cell index values generated by cell attachment. Cells are grown for 16–20 hours before ligand treatment. (2) Cell index values are obtained immediately following ligand treatment every 20 seconds for a total time of at least 100 minutes. (3) Cell index values are normalized by dividing by the cell index at the time of ligand addition and (4) baseline-corrected by subtracting the cell index obtained in vehicle-treated conditions. See Materials and Methods for more details. (TIF) [file pone.0029420.s009.tif]
